# Integrated physiologic, genomic and transcriptomic strategies involving the adaptation of allotetraploid rapeseed to nitrogen limitation

**DOI:** 10.1186/s12870-018-1507-y

**Published:** 2018-12-04

**Authors:** Zhen-hua Zhang, Ting Zhou, Qiong Liao, Jun-yue Yao, Gui-hong Liang, Hai-xing Song, Chun-yun Guan, Ying-peng Hua

**Affiliations:** 1 Southern Regional Collaborative Innovation Center for Grain and Oil Crops in China, College of Resources and Environmental Sciences, Hunan Agricultural University, Changsha, China; 2 National Center of Oilseed Crop Improvement, Hunan Branch, Changsha, China

**Keywords:** Allotetraploid rapeseed, Genomic selection, BnamiR827-*BnaNLA1s*-*BnaNRT1.7 s*, Nitrogen limitation adaptation, Nitrogen use efficiency, Transcriptional profiling

## Abstract

**Background:**

Nitrogen (N) is a macronutrient that is essential for optimal plant growth and seed yield. Allotetraploid rapeseed (A_n_A_n_C_n_C_n_, 2*n* = 4*x* = 38) has a higher requirement for N fertilizers whereas exhibiting a lower N use efficiency (NUE) than cereal crops. N limitation adaptation (NLA) is pivotal for enhancing crop NUE and reducing N fertilizer use in yield production. Therefore, revealing the genetic and molecular mechanisms underlying NLA is urgent for the genetic improvement of NUE in rapeseed and other crop species with complex genomes.

**Results:**

In this study, we integrated physiologic, genomic and transcriptomic analyses to comprehensively characterize the adaptive strategies of oilseed rape to N limitation stresses. Under N limitations, we detected accumulated anthocyanin, reduced nitrate (NO_3_^−^) and total N concentrations, and enhanced glutamine synthetase activity in the N-starved rapeseed plants. High-throughput transcriptomics revealed that the pathways associated with N metabolism and carbon fixation were highly over-represented. The expression of the genes that were involved in efficient N uptake, translocation, remobilization and assimilation was significantly altered. Genome-wide identification and molecular characterization of the microR827-*NLA1*-*NRT1.7* regulatory circuit indicated the crucial role of the ubiquitin-mediated post-translational pathway in the regulation of rapeseed NLA. Transcriptional analysis of the module genes revealed their significant functional divergence in response to N limitations between allotetraploid rapeseed and the model Arabidopsis. Association analysis in a rapeseed panel comprising 102 genotypes revealed that *BnaC5.NLA1* expression was closely correlated with the rapeseed low-N tolerance.

**Conclusions:**

We identified the physiologic and genome-wide transcriptional responses of oilseed rape to N limitation stresses, and characterized the global members of the BnamiR827-*BnaNLA1s*-*BnaNRT1.7s* regulatory circuit. The transcriptomics-assisted gene co-expression network analysis accelerates the rapid identification of central members within large gene families of plant species with complex genomes. These findings would enhance our comprehensive understanding of the physiologic responses, genomic adaptation and transcriptomic alterations of oilseed rape to N limitations and provide central gene resources for the genetic improvement of crop NLA and NUE.

**Electronic supplementary material:**

The online version of this article (10.1186/s12870-018-1507-y) contains supplementary material, which is available to authorized users.

## Background

Nitrogen (N) is a macronutrient that is essential for plant biomass and seed yield [1]. To achieve optimal growth and development, plants have to constantly acquire abundant N nutrients from soils. In agriculture, immense quantities of N fertilizers are applied worldwide annually to maintain crop productivity. This practice requires excessive amounts of energy and poses a remarkable threat to the environment. N use efficiency (NUE) is defined as the total biomass or grain yield produced per unit of applied fertilizer N [2], and improving NUE is critical for the favorable development of sustainable agriculture and ecosystem. In recent years, enhancement of plant N limitation adaptation (NLA) has shown to be an effective strategy to maintain or increase crop yields with reduced application of N fertilizers [2].

AtNLA1 is the first identified Really Interesting New Gene (RING)-type E3 ubiquitin ligase with the SYG1-Pho81-XPR1 (SPX) motif, and it functions as a positive regulator of the adaptability of *Arabidopsis thaliana* to N limitations [3]. AtNRT1.7/AtNPF2.13 is expressed mainly in the phloem of leaf minor veins and mediates the remobilization of excess NO_3_^−^ from the older leaves to younger ones [4]. AtNLA1 promotes the ubiquitin-mediated protein degradation of AtNRT1.7, which accelerates the source-to-sink remobilization of N nutrients [5]. AtNLA1 expression is repressed by N limitation mainly at the post-transcriptional level via the microRNA827 (miR827)-dependent regulation [5]. Thus, the miR827-*NLA1*-*NRT1.7* circuit plays a key role in the adaptability of plants to N limitations.

Oilseed rape (*Brassica napus* L.), a high-value staple crop species, is widely grown and harvested for the production of vegetable oil, livestock protein meal and biodiesel [6]. The allotetraploid *B. napus* (A_n_A_n_C_n_C_n_, ~ 1,345 Mb, 2*n* = 4*x* = 38) originates from spontaneous interspecific hybridization of the diploid progenitors *Brassica rapa* (A_r_A_r_, ~ 485 Mb, 2*n* = 2*x* = 20) [7] and *Brassica oleracea* (C_o_C_o_, ~ 630 Mb, 2*n* = 2*x* = 18) [8–10]. Compared with those in the model plant *A. thaliana* (~ 125 Mb, 2*n* = 2*x* = 10) (*Arabidopsis* Genome Initiative 2000) of Brassicaceae, the allopolyploidy events in *B. napus* generates many duplicated segments and homeologous regions, which further contribute to the formation of multi-copy gene families within the genome [9].

Unlike grain crops, *B. napus* has a relatively higher N nutrient requirement for optimal seed yield [11, 12]. Indeed, despite its strong NO_3_^−^ uptake capacity, oilseed rape shows the lowest NUE that has been known in crops [13]. This is because older leaves easily drop and detach from the plants before that N has been sufficiently remobilized to the sink organs [14, 15]. Therefore, strengthening the adaptability of oilseed rape to N limitations and avoiding early senescence of leaves, is essential for NUE enhancement. However, the central gene members that regulate NLA remain elusive in allotetraploid rapeseed because of its genome complexity. Thus, in this study, we were aimed to (i) identify the physiologic and transcriptomic responses of rapeseed plants to short-term and long-term N limitations; (ii) conduct genomic and transcriptional characterization of the core gene members of the miR827-*NLA1*-*NRT1.7* regulatory circuit, and (iii) propose the molecular strategies involving N limitation adaptation in allotetraploid rapeseed. Our genome-wide identification and molecular characterization of the BnamiR827-*BnaNLA1*-*BnaNRT1.7* circuit members indicated evolutionary conservation and functional divergence of the NLA regulatory mechanism between allotetraploid rapeseed and the model *Arabidopsis*. The transcriptomics-assisted gene co-expression network analysis of the NLA module would provide central gene resources for the genetic improvement of crop NLA and NUE.

## Results

### Physiologic responses of oilseed rape to N limitation

**Fig. 1:**
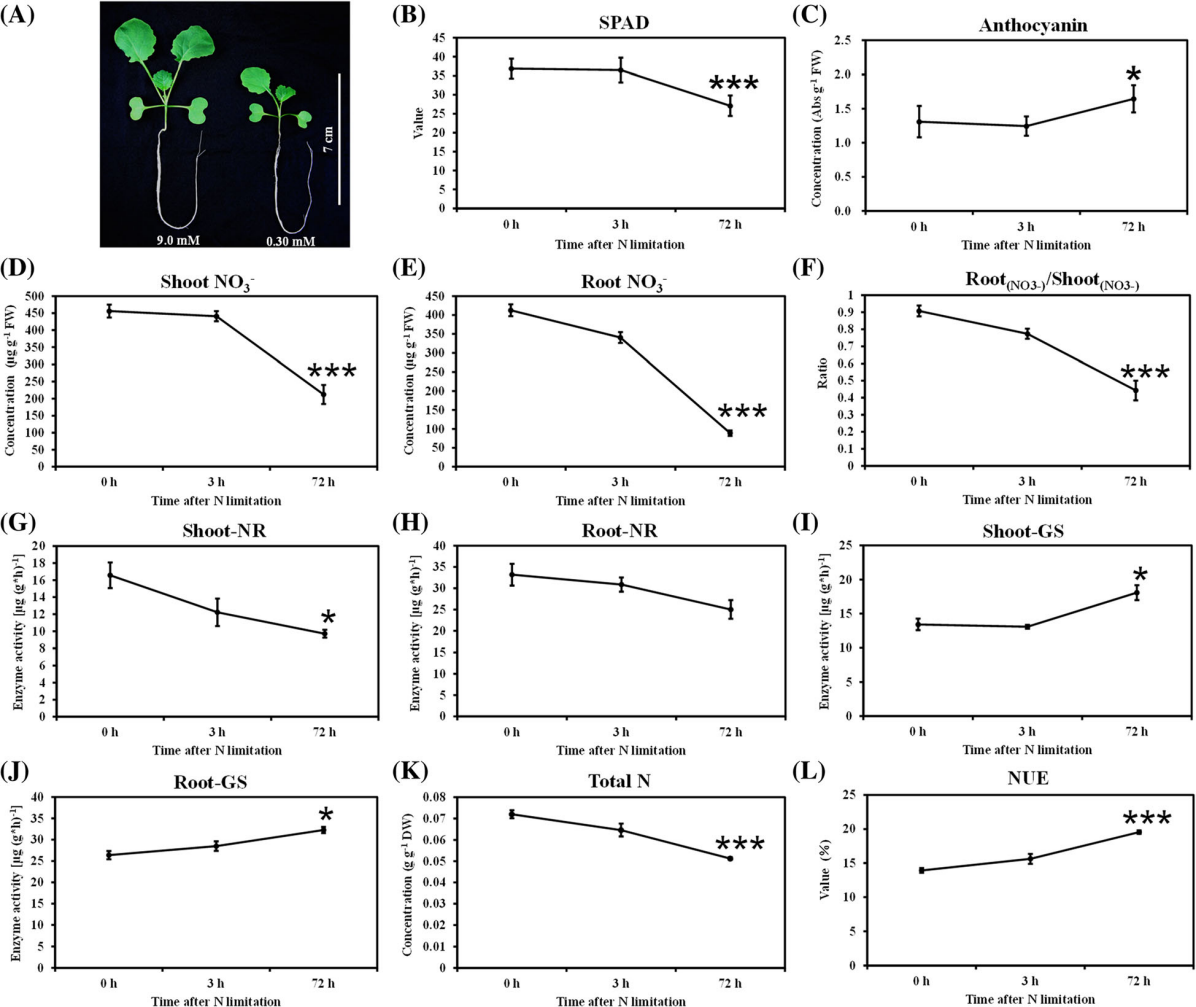
Physiologic responses of oilseed rape to nitrogen (N) limitation stresses. **a** Growth performance of the rapeseed plants (scale bar = 7 cm) that were hydroponically cultivated under high (9.0 mM) and low (0.30 mM) nitrate (NO_3_^−^) conditions for 10 d; (**b**) leaf SPAD values; (**c**) leaf anthocyanin concentrations; (**d**-**e**) NO_3_^−^ concentrations in the shoots (**d**) and roots (**e**); (**f**) ratio of shoot NO_3_^−^ concentrations to root NO_3_^−^ concentrations; (**g**-**h**) activity of NO_3_^−^ reductase (NR) in the shoots (**g**) and roots (**h**); (**i**-**j**) activity of glutamine synthetase in the shoots (**i**) and roots (**j**); (**k**) total N concentrations of the whole plants; (**l**) values of N use efficiency (NUE), NUE = total dry weight/total N content. For (**b**-**l**), the rapeseed plants that were grown under 9.0 mM NO_3_^−^ for 10 d were then transferred to 0.30 mM NO_3_^−^, and the shoots and roots were individually sampled at 0 h, 3 h and 72 h. Values denote means (*n* = 5), and error bars indicate standard error (SE) values. Significant difference was determined by one-way analysis of variance (ANOVA), which was followed by Tukey’s honestly significant difference (HSD) multiple comparison tests using the Statistical Productions and Service Solutions 17.0 (SPSS, Chicago, IL, USA). *: *p* < 0.05; **: *p* < 0.01; ***: *p* < 0.001

When NO_3_^−^ supply is insufficient, plants usually develop a set of adaptive responses to limited N growth conditions [2]. The physiologic responses of rapeseed to N limitation were determined by hydroponically growing the plants under high (9.0 mM) and low (0.30 mM) NO_3_^−^ conditions. After 10-d of plant growth, long-term N limitation severely inhibited the shoot and root growth of *B. napus*, which was indicated by smaller leaves (Fig. 1a). Moreover, the root volume (0.55 ± 0.09 cm^3^) of the rapeseed plants under N limitation was also significantly reduced than that (0.23 ± 0.04 cm^3^) under N sufficiency. Subsequently, the plant responses to short-term (3 h) and long-term (72 h) N limitation (0.30 mM) stresses were investigated in detail. Long-term limited N significantly reduced chlorophyll biosynthesis (Fig. 1b) and resulted in the over-accumulation of anthocyanin (Fig. 1c). Under severe N limitation, the ratio of root NO_3_^−^ concentration to shoot NO_3_^−^ concentration was significantly smaller than 1.0 (Fig. 1d-f), which indicated that the limited N nutrient resources were dominantly allocated to the shoots, which was less affected by N starvation than the roots, to facilitate the photosynthesis. The activity analyses of the N-metabolism associated enzymes revealed that the NR activity that was markedly reduced in the shoots did not significantly change in the roots (Fig. 1g, h), whereas the activity of glutamine synthetase was clearly elevated under N limitation (Fig. 1i, j). After exposure to low NO_3_^−^ conditions for 3 d, the plant biomass did not change significantly. The N concentrations of whole plants were markedly decreased with the duration of N limitation (Fig. 1k), whereas N depletion enhanced the NUE of rapeseed plants (Fig. 1l). Thus, compared with sufficient NO_3_^−^ supply, long-term, but not short-term, N limitation induced significant physiologic changes in the rapeseed plants.

### Genome-wide transcriptional responses of oilseed rape to N limitations

**Fig. 2:**
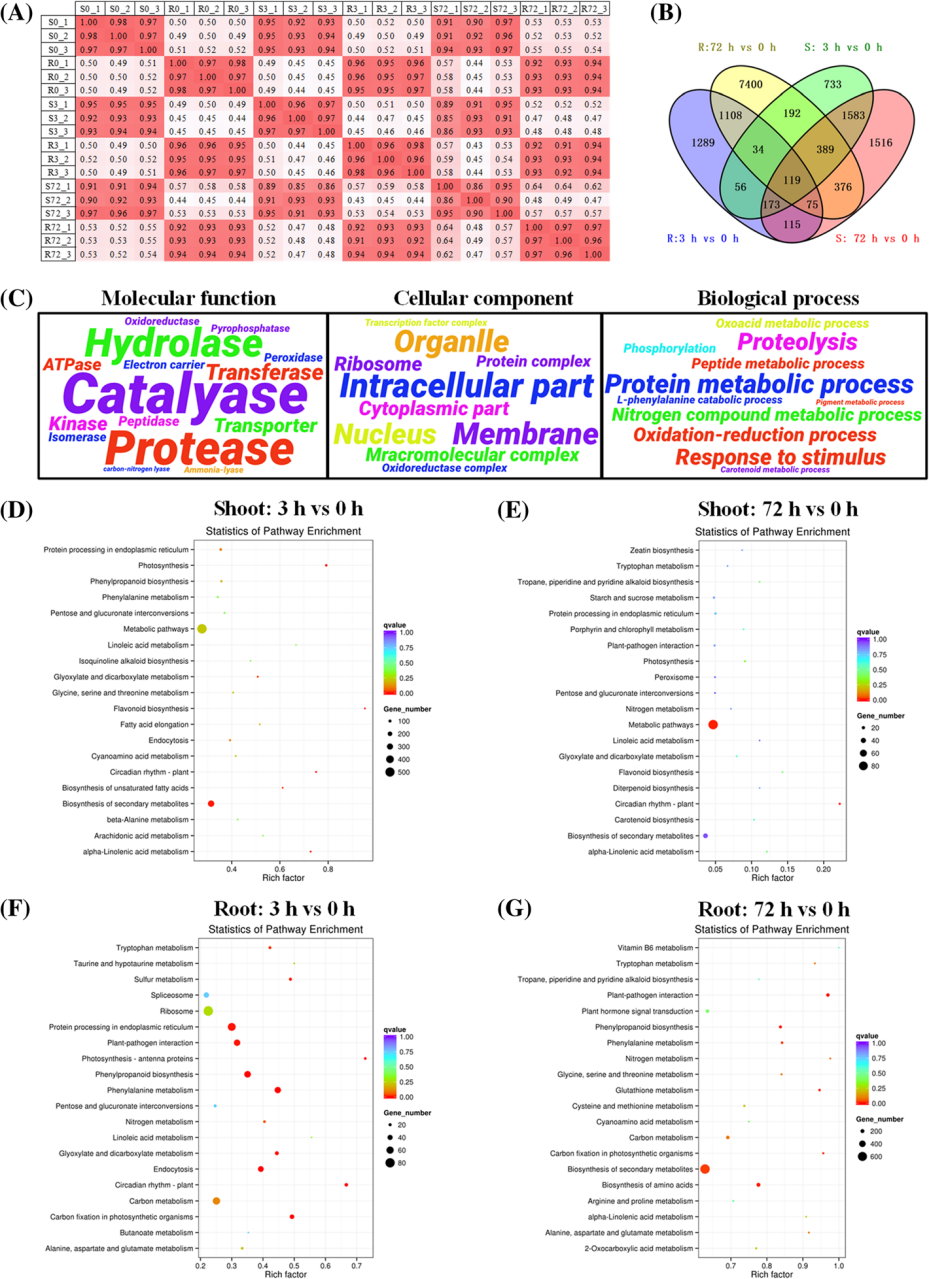
Genome-wide identification and characterization of the differentially expressed genes (DEGs) that were responsive to nitrogen (N) limitations. **a** *Pearson* correlation coefficients of the RNA-seq data between each pair of biological replicates. S and R indicate shoots and roots; S0/R0, S3/R3 and S72/R72 indicate shoots/roots at 0 h, 3 h and 72 h, respectively. **b**-**c** Venn diagram showing intersection analysis (**b**) and gene ontology (GO) term annotations of the DEGs. In the word cloud, the font sizes indicate the GO term numbers. The bigger the fonts are, the more the corresponding GO terms are. **d**-**g** KEGG enrichment analysis of the DEGs in the shoots (**d**, **e**) and roots (**f**, **g**) at 3 h and 72 h. The solid circle sizes represent the pathway enriched degree. The bigger the circles are, the more the corresponding KEGG items are. Regarding the RNA-seq experiment, the rapeseed plants that were grown under 9.0 mM NO_3_^−^ for 10 d were then transferred to 0.30 mM NO_3_^−^, and the shoots and roots were individually sampled at 0 h, 3 h and 72 h. False discovery rate (FDR) ≤ 0.05 and log_2_ (fold-change) ≥ 1 are used as the thresholds to identify DEGs

After discard of adapter sequences and low-quality reads, on average, approximately 5.0 **×** 10^7^ clean reads were obtained for each sample, and the total length of clean reads reached about 1.5 **×** 10^10^ nt with Q_20_ > 96% and Q_30_ > 92% (Additional file 1: Table S2). Most of the *Pearson* correlation coefficients were more than 0.90 between each pair of biological replicates (Fig. 2a), which indicated that the mRNA sequencing data were of good quality.

Subsequently, we detected the global gene differential expression profiling of *B. napus* under short-term and long-term N limitations compared with the sufficient N supply. In the shoots, a total of 3,279 and 4,346 genes were identified to be differentially expressed at 3 h and 72 h, respectively; in the roots, more DEGs were characterized, particularly at 72 h (Fig. 2b). An intersection analysis through a Venn diagram indicated that 119 DEGs were simultaneously detected in both the shoots and roots at 3 h and 72 h (Fig. 2b).

The GO enrichment analysis enabled us to characterize major biological functions of the DEGs under short-term and long-term N limitations. In this study, the GO terms were grouped into the three major categories: molecular function (MF), cellular component (CC), biological process (BP). Regardless of the shoots or the roots under both short-term and long-term N limitations, the most highly enriched GO term for CC was the intracellular part, whereas the catalase, protease and hydrolase were the three most over-represented enzymes in the MF category (Fig. 2c). In the BP annotations, the protein metabolism and proteolysis were the most two enriched items (Fig. 2c). To further identify the biological pathways that were active in *B. napus* during exposure to short-term and long-term N limitations, we characterized the pathways in which the DEGs were involved using the KEGG database. In the shoots at both 3 h and 72 h, the pathways for photosynthesis and flavonoid metabolism were highly enriched (Fig. 2d, e). In the roots, a large proportion of the DEGs were over-represented in the pathways involving the metabolism of phenylpropanoid, glutamine and carbon fixation, particularly at 72 h (Fig. 2f, g). Taken together, the integrated analysis of GO and KEGG indicated that carbon fixation (e. g. photosynthesis) and N metabolism (e. g. proteolysis) were strongly responsive to short-term or long-term N limitations.

### The role of anthocyanin in the adaptability of oilseed rape to N limitations

**Fig. 3:**
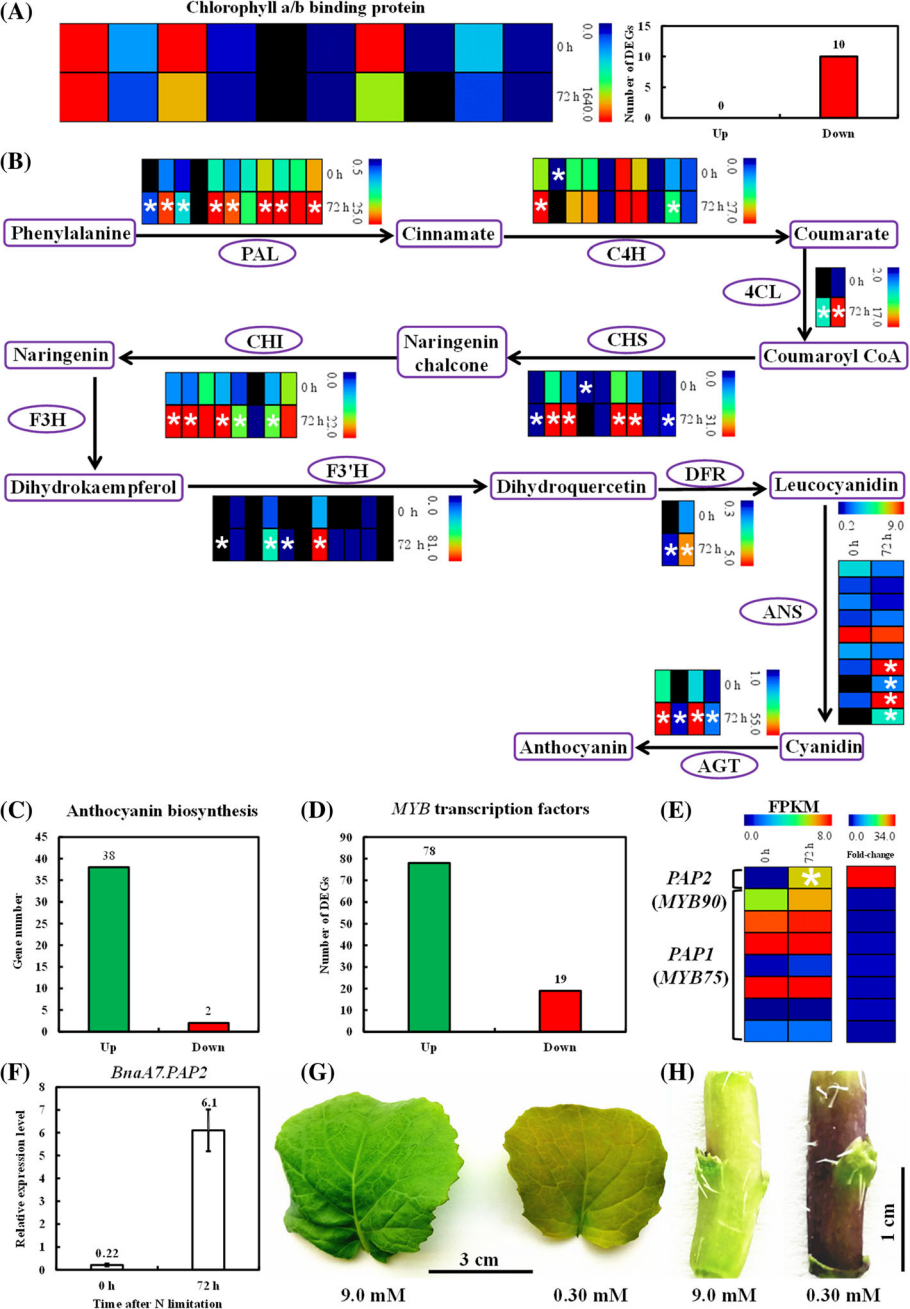
Transcriptional profiling of the phenylpropanoid pathway for the anthocyanin biosynthesis and rapeseed leaves/stems accumulating anthocyanin. **a**, **b** Transcriptional profiling of the chlorophyll-binding protein genes (**a**) and the phenylpropanoid pathway for the anthocyanin biosynthesis (**b**) in the shoots under sufficient N supply (0 h) and long-term N limitation (72 h) conditions. Enzymes in each step: PAL, phenylalanine ammonia lyase; C4H, cinnamic acid 4-hydroxylase; 4CL, coumaroyl-CoA synthase; CHS, chalcone synthase; CHI, chalcone-flavanone isomerase; F3H, flavanone 3-hydroxylase; F3’H, flavanone 3′-hydroxylase; DRF, dihydroflavonol 4-reductase; ANS, anthocyanidin synthase; AGT, anthocyanin glycosyltransferase. Each column indicates a gene. **c**, **d** Number of the differentially expressed genes (DEGs) involved in the anthocyanin biosynthesis (**c**) and MYB transcription factors (**d**). Up, up-regulation; down: down-regulation; n.s., not significant. **e** Transcriptional profiling of the *Production of Anthocyanin Pigment* (*PAP*) genes *BnaPAP1s* (*BnaMYB75s*) and *BnaA7.PAP2* (*BnaA7.MYB90*) under long-term N limitations. **f** Relative expression of *BnaA7.PAP2* under N limitations by the qRT-PCR assay. Heat maps of gene expression profiling were generated using Multi-experiment Viewer (Mev, http://www.mybiosoftware.com/mev-4-6-2-multiple-experiment-viewer.html). False discovery rate (FDR) R) ultiown-log_2_(fold-change) ≥ 1 were used as the thresholds to identify DEGs. Regarding the RNA-seq experiment and qRT-PCR assays, the rapeseed plants that were grown under 9.0 mM NO_3_^−^ for 10 d were then transferred to 0.30 mM NO_3_^−^, and the shoots and roots were individually sampled at 0 h, 3 h and 72 h, respectively. The color scales of heat maps indicate the expression levels (FPKM values) or fold-changes of gene expression, and the differentially expressed genes between the control (0 h) and the long-term N limitation treatment (72 h) are indicated by asterisks. (**g**, **h**) Rapeseed leaves (**g**) and stems (**h**) with accumulated anthocyanin. The rapeseed plants that were hydroponically cultivated under high (9.0 mM) NO_3_^−^ condition for 10 d were then transferred to 9.0 mM and 0.30 mM NO_3_^−^ conditions for 10 d

Anthocyanins, important secondary metabolites in plants, protect senescing leaves from photo-damages; moreover, they also promote the efficient remobilization of nutrients (especially N) within the plants [14]. In this study, we found that the genes that are involved in the biosynthesis of chlorophyll pigments were significantly down-regulated under N limitation (Fig. 3a), suggesting the serious degradation of chlorophyll. Anthocyanins are produced mainly through the phenylpropanoid-dependent pathway, as presented in Fig. 3b. This biosynthesis begins with phenylalanine that then were converted into cinnamic acid catalyzed by phenylalanine ammonia lyase (PAL), and disintegrates into several branches at coumaroyl CoA. In the flavonoid route, where chalcone synthase (CHS) catalyzes the flavonoid formation derived from coumaroyl CoA, and then contributes to the production of flavonol, cyanidin, and anthocyanin (Fig. 3b).

Under severe N limitation, the anthocyanin concentrations increased markedly in the rapeseed leaves (Fig. 1c). Further, we investigated the transcriptional fingerprints of the genes that are involved in anthocyanin biosynthesis under N limitation. The results showed that 95% of the DEGs were significantly up-regulated under limited N supply (Fig. 3c). The MYB transcription factors, particularly the MYB75 (Production of Anthocyanin Pigment 1, PAP1) and MYB90 (PAP2) that mediates the anthocyanin biosynthesis, are shown to play positive roles in the plant responses to N limitations [15–17]. Among the genome-wide DEGs of *BnaMYBs*, we found that a major proportion (80%) of them were induced by N limitation (Fig. 3d). Both the RNA-seq and qRT-PCR results showed that the transcript level of *BnaA7.PAP2* was remarkably higher under N limitation than under sufficient N supply (Fig. 3e-f). It indicated the dominant roles of MYBs in the anthocyanin biosynthesis-mediated adaptation of rapeseed to N limitation stresses. After exposure to long-term N limitation, the rapeseed plants accumulated abundant anthocyanin in the leaves and stems (Fig. 3g, h). Indeed, the stem anthocyanin was observed shortly after N limitation, which can be potentially used as an indicator for diagnosis of crop N nutrient status and identification of the rapeseed genotypes with differential adaptabilities to N limitations.

### Transcriptional responses of the genes associated with N transport and metabolism to N limitations

**Fig. 4:**
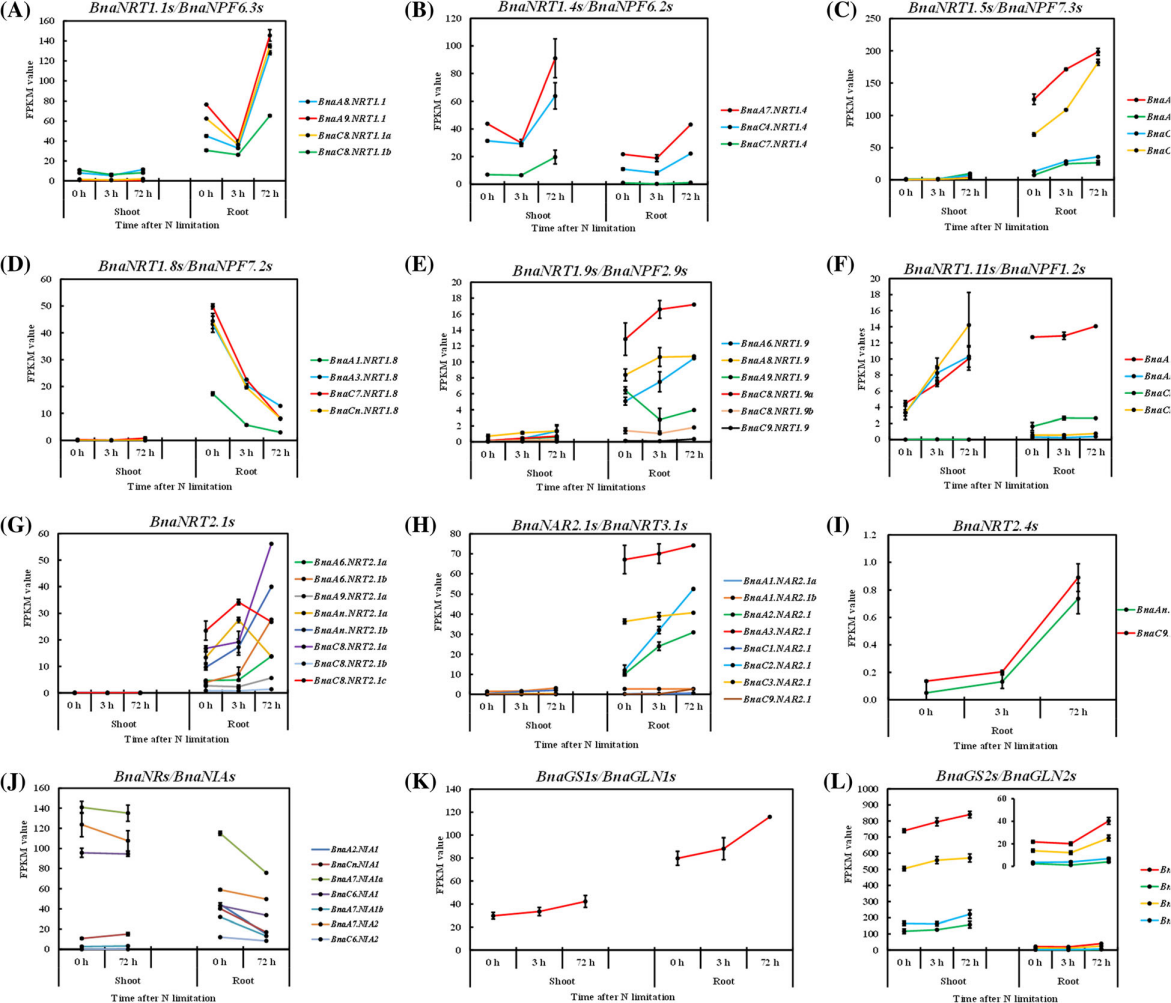
Transcriptional profiling of the genes associated with nitrogen (N) transport and metabolism under short-term (3 h) and long-term (72 h) N limitations. Expression profiling of *BnaNRT1.1 s/BnaNPF6.3 s* (**a**), *BnaNRT1.4 s/BnaNPF6.2 s* (**b**), *BnaNRT1.5 s/BnaNPF7.3 s* (**c**), *BnaNRT1.8 s/BnaNPF7.2 s* (**d**), *BnaNRT1.9 s/BnaNPF2.9 s* (**e**), *BnaNRT1.11 s/BnaNPF1.2 s* (**f**), *BnaNRT2.1 s* (**g**), *BnaNAR2.1 s/BnaNRT3.1 s* (**h**), *BnaNRT2.4 s* (**i**), *BnaNRs/BnaNIAs* (**j**), *BnaGS1s/BnaGLN1s* (**k**) and *BnaGS2s/BnaGLN2s* (**l**) in the shoots and roots. The rapeseed plants that were grown under 9.0 mM NO_3_^−^ for 10 d were then transferred to 0.30 mM NO_3_^−^, and the shoots and roots were individually sampled at 0 h, 3 h and 72 h. For each diagram, the *x*-axis indicates the time (h) after N limitations imposing on rapeseed plants, and the *y*-axis shows gene expression levels (FPKM values) obtained from RNA-seq. Values denote means (*n* = 3), and error bars indicate standard error (SE) values

Among the numerous DEGs, we first paid much more attention to the genes that are implicated in efficient N uptake, transport and N assimilation; these genes are crucial for the adaptive responses of plants to N limitations [18]. Our transcriptomics results showed that *BnaNRT1.1 s/BnaNPF6.3 s* were strongly induced in the roots of rapeseed plants exposed to 72-h N limitation (Fig. 4a), and they might contribute to efficient N influx into the root cells. Different from *AtNRT1.4/AtNPF6.2*, whose transcript level is not affected by NO_3_^−^ supply levels [19], the mRNA abundances of *BnaNRT1.4 s/BnaNPF6.2 s* were markedly elevated in the shoots and roots by severely limited N (Fig. 4b) and they might be favorable for efficient N storage in petioles. Similar to *AtNRT1.5/AtNPF7.3*, the four *BnaNRT1.5 s/BnaNPF7.3 s* were also expressed preferentially in the roots and they were obviously up-regulated under both short-term and long-term N limitations (Fig. 4c). In contrast, the four *BnaNRT1.8/BnaNPF7.2* genes were strongly repressed in the roots by N limitations (Fig. 4d). The transcript abundances of the *NRT1* member facilitating NO_3_^−^ loading into the root phloem, *BnaNRT1.9 s/BnaNPF2.9 s*, also increased under limited N supply (Fig. 4e). Combining the expression profiling of *BnaNRT1.5 s*, *BnaNRT1.8 s* and *BnaNRT1.9 s*, we proposed that more N was preferentially allocated to the shoots, which coincided with the result shown in Fig. 1f. Three of the *BnaNRT1.11 s/BnaNPF1.2 s*, potentially involved in xylem-to-phloem transfer for redistributing NO_3_^−^ into developing leaves [20], were greatly induced in the shoots, whereas *BnaA10.NRT1.11* also showed higher expression levels in the roots under N deficiency (Fig. 4f).

In terms of the high-affinity NO_3_^−^ transporters, we focused on the main regulator *BnaNRT2.1 s* and their partners *BnaNAR2.1 s/BnaNRT3.1 s*. The general expression profiling of both *BnaNRT2.1 s* and *BnaNAR2.1 s* showed that their expression levels were increased in the roots by insufficient N supply (Fig. 4g, h). Additionally, NRT2.4 and NRT2.5 are also implicated in high-affinity N uptake [21, 22]. Both of the two *BnaNRT2.4* family homologs were significantly up-regulated in the roots whereas *BnaNRT2.5 s* showed very smaller FPKM values although they were induced by N deficiency (Fig. 4i).

In addition to the expression alterations of genes implicated in efficient N uptake and allocation, the transcriptional changes of the N-metabolism genes were also observed (Fig. 4j-l). With the decrease in external N supply, the NR genes *BnaNIA1s* and *BnaNIA2s* were down-regulated in the shoots and roots (Fig. 4j), which is consistent with the reduced enzyme activity (Fig. 1g, h). In contrast, the expression of both *BnaGS1s/BnaGLN1s* and *BnaGS2s/BnaGLN2s* was induced in both the shoots and roots (Fig. 4k-l). The integrated analysis of expression profiling and enzyme activity of GS (Fig. 1i, j) indicated that the enhanced assimilation of inorganic N into amino acids might be helpful for the adaptability of rapeseed plants to N limitations.

### Global identification and molecular characterization of *BnaNLAs*

The miR827-*NLA1*-*NRT1.7* regulatory circuit functions as a pivotal pathway involving the adaptive responses of plants to N limitations [5]. Therefore, we focused on the identification and characterization of the roles of the miR827-*NLA1*-*NRT1.7* regulatory pathway in the adaptive strategies of oilseed rape to N limitation stresses.

To compare the evolutionary diversity of the *NLA* genes among various plant species, we retrieved *NLAs* in 22 plant species, including 19 dicots, and three monocots (Additional file 1: Figure S1). In general, the copy number of the *NLA* genes was not closely correlated with the genome sizes. We found that, relative to that in the other plant species, the allotetraploid *B. napus* had the largest *NLA* gene family, including four *BnaNLA1s* and four *BnaNLA2s* (Additional file 1: Figure S1). Moreover, the number of the *NLA* genes in *B. napus* was equal to the *NLA* gene sum in *B. rapa* and *B. olereacea* (Additional file 1: Figure S1), which implied that all the *NLAs* were maintained during the allopolyploidy process. The genomic organization analysis showed that both *NLA1* and *NLA2* subfamily genes in *B. napus* might have largely expanded mainly through segmental duplication (Additional file 1: Figure S2). Phylogeny analysis confirmed that the BnaNLA proteins can be grouped into two subfamilies, namely, BnaNLA1s and BnaNLA2s (Additional file 1: Figure S3A), both of which experienced strong purifying/negative (Ka/Ks < 1.0) pressure selection (Additional file 1: Table S3) in order to preserve gene function. The DIVEGE analysis showed that the type II coefficient θ_II_ ± SE was > 0 (Additional file 1: Figure S3B), which indicated that obvious functional divergence had occurred between the BnaNLA1 and BnaNLA2 subgroup proteins. The segregation of *Arabidopsis* and *Brassica* plants might have occurred 12–20 million years ago (Mya) [23–25]. The results showed that *BnaNLAs* might have diverged from the corresponding homologs in *Arabidopsis* approximately 11.3–18.0 Mya, which implied that plant speciation was accompanied by the divergence of the *BnaNLA* family genes.

Previous studies have shown that NLA1 plays a key role in the regulation of plant adaptive responses to N limitations [5]. The NLA1s of dicots and monocots, all of which were identified to have microRNA827 (miR827) binding sites, were phylogenetically categorized into two clusters, and it implied that the NLA1 proteins divergence occurred after organism speciation (Additional file 1: Figure S3C). The four *BnaNLA1s* that encode approximately 300 amino acids were physically mapped onto four chromosomes (A_n_ sub-genome: A_n_9 and A_n_10; C_n_ sub-genome: C_n_5 and C_n_8) of *B. napus*, all of which were located in the A chromosomal block of the least fractionated genome (Additional file 1: Table S4). The computed molecular weights of the NLA proteins were close to 38.0 kDa except for BnaA4.NLA2, and their pIs were approximately 8.5 (Additional file 1: Table S4). The majority of the NRT2 protein instability indices (IIs) were > 40.0, and the NLA family members that were hydrophilic had the GRAVY values that ranged from − 0.421 (BnaA10.NLA1) to − 0.233 (BnaA4.NLA2) (Additional file 1: Table S4).

**Fig. 5:**
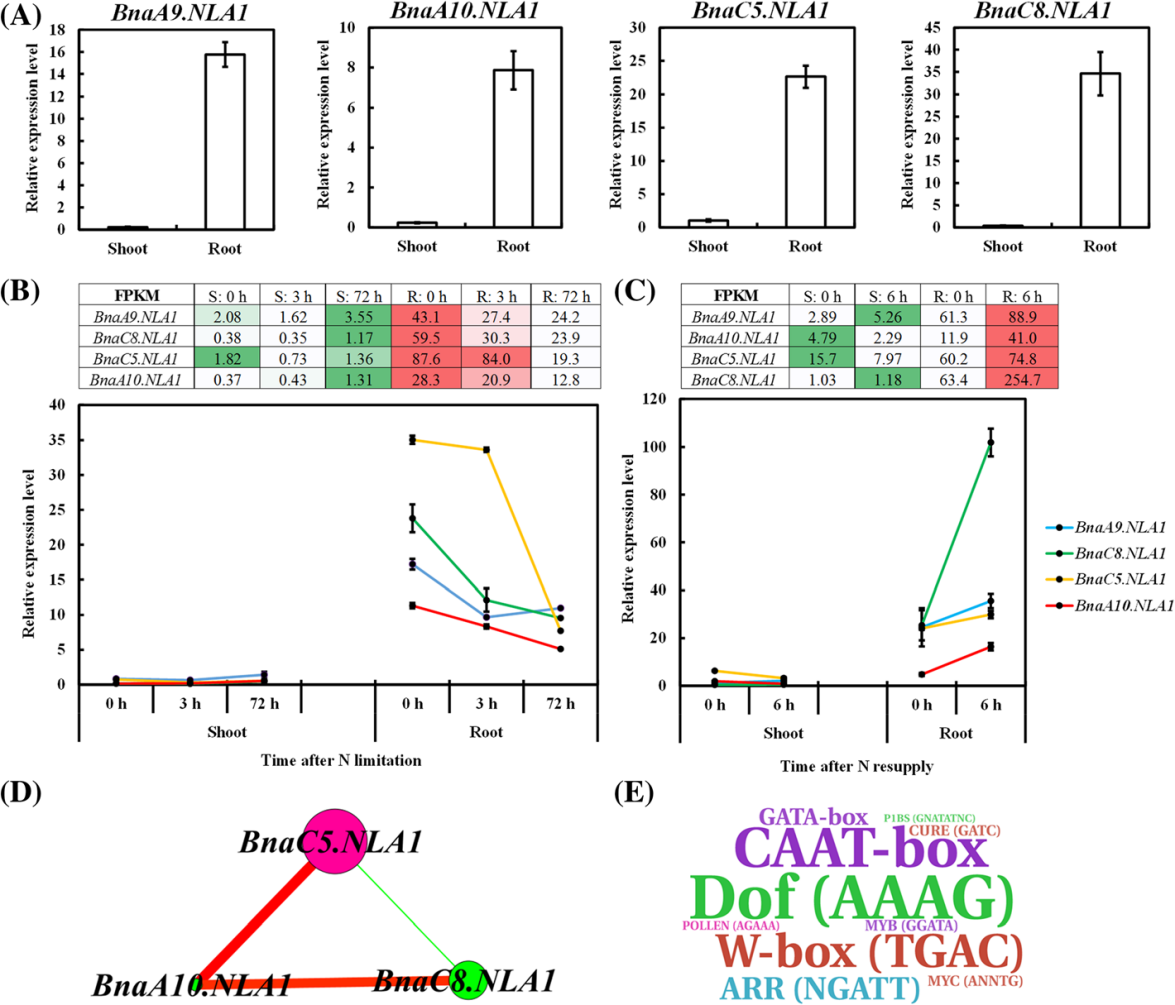
Molecular characterization of the expression pattern and transcriptional responses of *BnaNLA1s* to different N supply levels. **a** The qRT-PCR assay results showing the expression pattern of *BnaNLA1s*. **b**, **c** Transcriptional profiling of *BnaNLA1s* to N limitations (**b**) and N resupply (**c**). The heat maps show the mRNA levels (FPKM values) of *BnaNLA1s* that were identified by the transcriptome sequencing, and the curve diagram present the relative expression levels of *BnaNLA1s* that were validated by qRT-PCR assays. Regarding the NO_3_^−^-depletion treatments, the rapeseed seedlings that were cultivated under high NO_3_^−^ (9.0 mM) for 10 d were then transferred to low NO_3_^−^ (0.30 mM). At 0 h, 3 h and 72 h, the shoots and roots of the seedlings were individually sampled. Reagrding the NO_3_^−^ resupply treatments, the *B. napus* seedlings that were hydroponically cultivated under high NO_3_^−^ (9.0 mM) for 9 d were then transferred to NO_3_^−^-free solution for 3 d. The seedlings were sampled after being treated with 9.0 mM NO_3_^−^ for 6 h, respectively. Values denote means (*n* = 3), and error bars indicate standard error (SE) values. **d** Gene co-expression network analysis of *BnaNLA1s*. Cycle nodes represent genes, and the size of the nodes represents the power of the interrelation among the nodes by degree values. Edges between two nodes represent the interactions between genes. **e** Identification of the putative *cis*-acting regulatory elements (CREs) of the 2.0-kb genomic sequences upstream the start codon (ATG) of *BnaNLA1s*. Over-representation of the CREs in the gene promoters, which is delineated by the WordArt program. The bigger the font size, the more the CREs

Similar to the E3 ubiquitin ligase AtNLA1, both BnaNLA1s and BnaNLA2s contained an N-terminal SPX domain and a C-terminal RING domain in addition to other conserved motifs (Additional file 1: Figure S3D, E), and subcellular localization predicted that they were localized on the plasma membrane. To determine the roles of the *BnaNLA* family genes in the regulation of rapeseed plants to N limitations, we investigated their expression pattern and transcriptional responses to different N supply levels. In terms of the *BnaNLA1* subfamily, the qRT-PCR assay results showed that all the four members were expressed predominantly in the roots rather than in the shoots (Fig. 5a). In *A. thaliana*, *AtNLA1* is not regulated by N supply changes at the transcriptional level [5]. Interestingly, we found that all the *BnaNLA1s* were transcriptionally down-regulated by limited N supply (Fig. 5b) and were up-regulated by N resupply (Fig. 5c), which potentially implied that their different regulatory pathways differed from that in the model *Arabidopsis*. Based on the expression profiling of the *BnaNLA1* family genes, we constructed a gene co-expression network, and identified *BnaC5.NLA1* as the central member (Fig. 5d), and it was assumed to play a core role in the adaptive responses of rapeseed to N limitation stresses. Considering the existing transcriptional responses of *BnaNLA1s* to N limitations, we investigated the CREs in the gene promoters that were involved in the transcriptional regulation of *BnaNLA1s*. We found that the binding sites of the DNA with one finger (Dof), GATA-box, W-box (TGAC) and MYB TFs were highly enriched in the promoters (Fig. 5e), most of which have proved to be involved in the molecular response of plants to N status [26–28]. Among these, the binding sites of the Dof proteins were the highest over-represented (Fig. 5e), which implied that the Dof TFs might play key roles in the transcriptional regulation of the *BnaNLA1* family genes.

In *Arabidopsis*, the role of AtNLA2 has been elusive. In *B. napus*, although four *NLA2* members were annotated in the genome, however, we only identified the expression of *BnaA4.NLA2* and *BnaC4.NLA2* through qRT-PCR and RNA-seq assays. Similar to *BnaNLA1s*, *BnaNLA2s* were also expressed mainly in the roots of rapeseed plants (Additional file 1: Figure S4A). However, the patterns of their transcriptional responses to different N supply were opposite to those of *BnaNLA1s*. Under limited N supply, the expression of *BnaNLA2s* was up-regulated (Additional file 1: Figure S4B) whereas their transcript levels were repressed by N resupply (Additional file 1: Figure S4C).

### Molecular characterization of BnamiR827

The *NLA1* gene has been reported to be a target of miR827 in *A. thaliana*, and miR827-mediated NLA repression is shown to play a key role in the adaption of plants to N limitations [5]. Previous studies have identified that the miR827 family has only one member in allotetraploid *B. napus* through BLAST analysis and high-throughput degradome sequencing [29, 30].

**Fig. 6:**
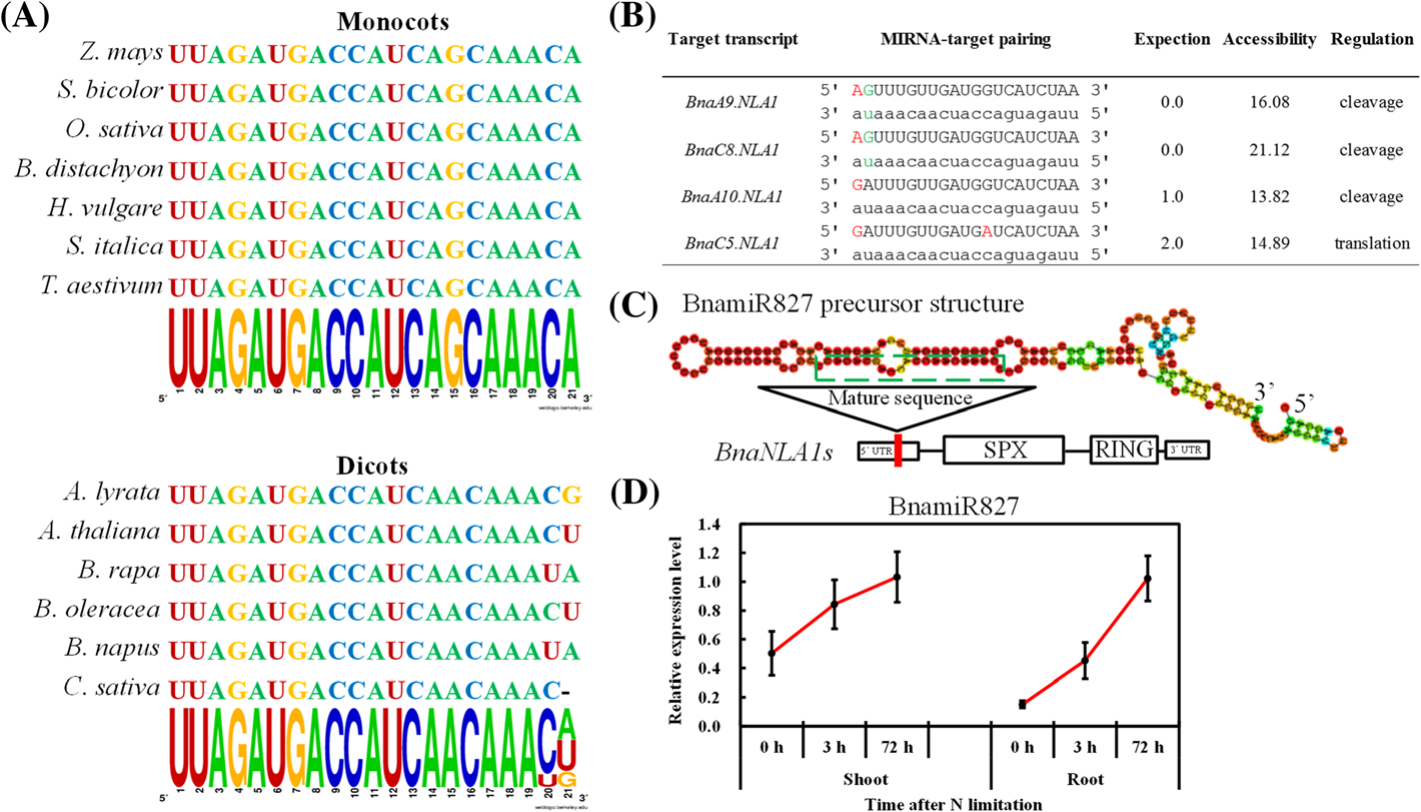
Molecular identification and characterization of miR827 in *Brassica napus*. **a** Multiple alignment of mature miR827 sequences in monocot and dicot species. **b** The target genes of BnamiR827in the allotetraploid rapeseed genome. Mismatch nucleotides are indicated in red, and the G-U pair is denoted by green. The BnamiR827 and its target gene sequences are shown with lowercase and capital letters, respectively. **c** The secondary structure of the precursor sequence of BnamiR827. The mature sequence of BnamiR827 is boxed by dashed lines, and the vertical red line in the 5′ untranslated region of *BnaNLA1s* indicates the BnamiR827 target site. **d** Relative expression of BnamiR827 under short-term and long-term nitrogen (N) limitations. The rapeseed seedlings that were cultivated under high nitrate (NO_3_^−^) (9.0 mM) for 10 d were then transferred to low NO_3_^−^ (0.30 mM). At 0 h, 3 h and 72 h, the shoots and roots of the seedlings were individually sampled. Values denote means (*n* = 3), and error bars indicate standard error (SE) values

Multiple sequence alignment showed that miR827 is highly conserved in both monocot and dicot species only with two nucleotide variations in the 3′-end of dicot miR827s (Fig. 6a). To identify the target preference of miR827 in the genome (A_n_A_n_C_n_C_n_) of allotetraploid rapeseed, we submitted the BnamiR827 sequence to the psRNATarget online program, a plant small-RNA target analysis server [31]. In rice, no target site of miR827 was found along the sequence of the *OsNLA* transcript [32], whereas four *BnaNLA1* members were identified to be the targets of BnamiR827 (Fig. 6b). The mRNA cleavage by BnamiR827 was predicted to occur in three *BnaNLA1* genes (*BnaA9.NLA1*, *BnaA10.NLA1* and *BnaC8.NLA1*) except *BnaC5.NLA1*, which was potentially repressed at the translational level by BnamiR827 (Fig. 6b). Further, we determined that BnamiR827 potentially could potentially bind to the 5′-end untranslated regions of *BnaNLAs* (Fig. 6c). To further understand the transcriptional responses of BnamiR827 to short-term and long-term N limitations, we tested its expression levels through qRT-PCR assays. The results showed that, irrespective of in the shoots or the roots, the expression of BnamiR827 was up-regulated by N limitations (Fig. 6c), which was opposite to the expression pattern of *BnaNLA1s* (Fig. 5b).

### Genome-scale identification and molecular characterization of *BnaNRT1.7 s*

In the rapeseed genome, we identified six *NRT1.7* homologs, encoding approximately 600 hydrophobic amino acids (Additional file 1: Table S5), which are distributed on four chromosomes (A_n_ sub-genome: A2 and A7; C_n_ sub-genome: C6 and C7) (Additional file 1: Figure S5). Phylogeny analysis revealed that the *NRT1.7* genes in *B. napus* were derived from their corresponding homologs in the diploid progenitors *B. rapa* and *B. oleracea* (Additional file 1: Figure S6A). Analysis of nucleotide substitution rates of *BnaNRT1.7 s* showed that they had experienced strong negative selection, and diverged from the corresponding *Arabidopsis* homologs approximately 14.3–15.7 Mya (Additional file 1: Figure S6B) when plant speciation was accompanied by the divergence of the *BnaNRT1.7* family genes. The conserved motif analysis suggested high similarities among the *BnaNRT1.7* family members (Additional file 1: Figure S6C-D), and all of them were predicted to be localized on the plasma membrane with 12 transmembrane regions (Additional file 1: Table S5).

**Fig. 7:**
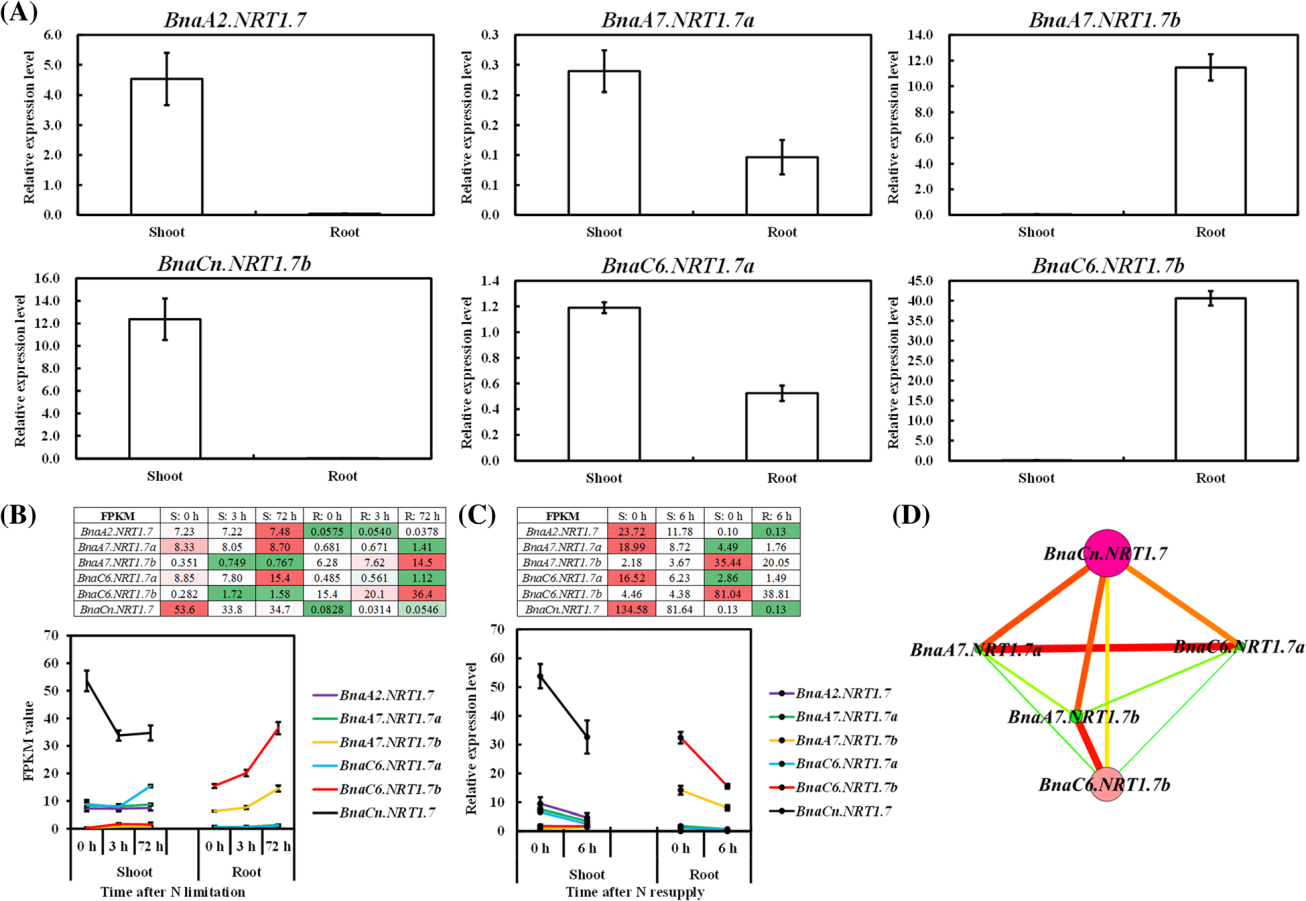
Molecular characterization of the expression pattern and transcriptional responses of *BnaNRT1.7 s* to different N supply levels. **a** The qRT-PCR assay results showing the expression pattern of *BnaNRT1.7 s*. **b**, **c** Transcriptional responses of *BnaNRT1.7 s* to N limitations (**b**) and N resupply (**c**). The heat maps show the mRNA levels (FPKM values) of *BnaNLA1s* that were identified by the transcriptome sequencing, and the curve diagram present the relative expression levels of *BnaNLA1s* that were validated by qRT-PCR assays. Regarding the NO_3_^−^-depletion treatments, the seedlings that were cultivated under high NO_3_^−^ (9.0 mM) for 10 d were then transferred to low NO_3_^−^ (0.30 mM). At 0 h, 3 h and 72 h, the shoots and roots of the seedlings were individually sampled. Regarding the NO_3_^−^ resupply treatments, the *B. napus* seedlings that were hydroponically cultivated under high NO_3_^−^ (9.0 mM) for 9 d were then transferred to NO_3_^−^-free solution for 3 d. The seedlings were sampled after being treated with 9.0 mM NO_3_^−^ for 6 h, respectively. Values denote means (*n* = 3), and error bars indicate standard error (SE) values. **d** Gene co-expression network analysis of *BnaNRT1.7 s*. Cycle nodes represent genes, and the size of the nodes represents the power of the interrelation among the nodes by degree values. Edges between two nodes represent the interactions between genes

To determine the roles of the *BnaNRT1.7* family genes in the regulation of rapeseed plants to N limitations, we investigated their expression pattern and transcriptional responses to different N supply levels. In *A. thaliana*, *NRT1.7* is expressed mainly in the phloem of the leaf minor vein [4]. However, the qRT-PCR assay results showed that four of the family members were expressed predominantly in the shoots, except *BnaA7.NRT1.7b* and *BnaC6.NRT1.7b* that were clustered in the same phylogenetic clade (Additional file 1: Figure S6A) were expressed preferentially in the roots (Fig. 7a). Further, *BnaCn.NRT1.7* and *BnaA7.NRT1.7b/BnaC6.NRT1.7b* that were up-regulated by long-term N limitation, were repressed by N resupply in the shoots and roots, respectively (Fig. 7b, c). Based on the expression profiling of the *BnaNRT1.7* family genes, we constructed a gene co-expression network. Further, we identified that *BnaCn.NRT1.7* and *BnaC6.NRT1.7b* were the central members (Fig. 7d), which were proposed to play core roles in the phloem N remobilization of both the shoots and roots under limited N stresses, respectively.

### Natural variations in the BnamiR827-*BnaNLA1-BnaNRT1.7* expression among rapeseed genotypes

**Fig. 8:**
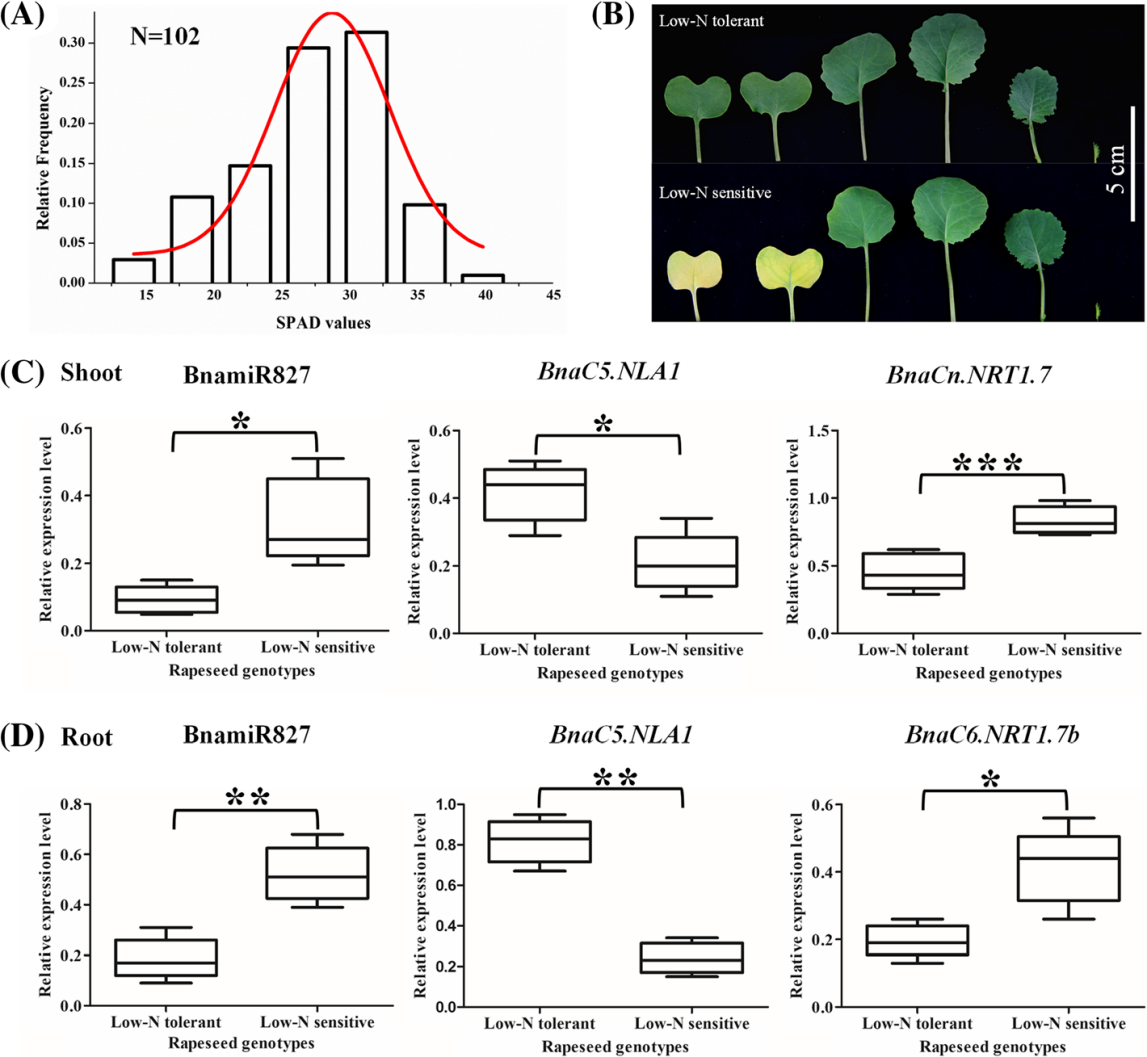
Differential physiologic and molecular responses to nitrogen (N) limitation in allotetraploid rapeseed genotypes. **a** Relative frequency of SPAD values in a rapeseed panel comprising 102 genotypes under limited N supply. The rapeseed plants that were grown under sufficient N (9.0 mM) condition were then transferred to limited N (0.30 mM) for 7 d, and the SPAD values of their mature leaves were assayed. **b** Differences in growth performance of the low-N-tolerant and low-N-sensitive genotypes that were identified from the 102 rapeseed accessions. Scale bar: 5 cm. **c**, **d** The qRT-PCR assay results of the BnamiR827-*BnaC5.NLA1*-*BnaC6.NRT1.7b /BnaC6.NRT1.7b* regulatory circuit in the shoots (**c**) and roots (**d**). The rapeseed plants that were grown under sufficient N (9.0 mM) condition were then transferred to limited N (0.30 mM) for 3 d. Five extremely low-N-tolerant genotypes and five extremely low-N-sensitive genotypes were selected, and the shoots and roots were individually sampled for the qRT-PCR assays. Significant difference was determined by two-tailed paired *t*-test. *: *p* < 0.05; **: *p* < 0.01; ***: *p* < 0.001

To further understand the roles of the BnamiR827-*BnaNLA1*-*BnaNRT1.7* regulatory circuit in the adaptation of rapeseed to N limitations, we conducted a comparative transcriptional analysis of the pathway. In a rapeseed panel comprising 102 accessions under limited N supply, we found that the SPAD values of the mature leaves were normally distributed and had a coefficient of variation of 32.6% (Fig. 8a), which indicated that wide variations in N limitation adaptation occurred among the rapeseed genotypes. Compared with the low-N tolerant rapeseed genotypes, the low-N sensitive rapeseed genotypes showed obvious early senescence of the mature leaves that was induced by N limitations (Fig. 8b). Further, among the 102 rapeseed genotypes, five accessions with extreme low-N tolerance and five with extreme low-N sensitivity were selected, respectively, and they were used to determine the regulation of the BnamiR827-*BnaNLA1*-*BnaNRT1.7* regulatory module in the differential responses to N limitations between the rapeseed genotypes. In both the shoots and roots, higher expression of *BnaC5.NLA1* and lower transcript levels of BnamiR827 and *BnaCn.NRT1.7/BnaC6.NRT1.7b* were identified in the low-N tolerant genotypes than in the low-N tolerant genotypes (Fig. 8c, d). It indicated that excessive expression of *BnaNRT1.7 s* induced remarkable remobilization of N from source to sink organs, which decreased the adaptability of rapeseed plants to N limitation stresses.

## Discussion

### Physiologic and transcriptional characterization of oilseed rape to N limitations

Unlike cereals, *B. napus* has a relatively higher nutrient requirement for optimal plant growth and seed yield [11], although it is hypersensitive to N limitation conditions. Strengthening the adaptation of rapeseed to N limitation is important for current agriculture production, in which excessive N fertilizers are routinely applied to increase seed yield worldwide [12]. Because 50–70% of the applied N cannot be absorbed by crops, excessive use of N fertilizers inevitably increases the cost of crop production as well as leads to environmental pollution [33]. One effective way to overcome these shortcomings is to genetically improve the adaptability of crops to N limitation, which requires the elucidation of the physiologic and molecular mechanism underlying NLA [2].

The physiologic and biochemical changes involved in the adaptation of rapeseed plants to N limitations include the reduction of growth and photosynthesis (Fig. 1a, b), the accumulation of abundant photodamage-protecting anthocyanins (Fig. 1c), elevation of N translocation from roots to shoots (Fig. 1d-f) and N assimilation enzyme activity (Fig. 1i, j). Moreover, we found that the rapeseed NUE was significantly elevated under N limitations (Fig. 1l), which indicated that improving the adaptability of crop species to limited N might be an effective strategy for NUE enhancement in agriculture production. Consistent with the physiologic data, the high-throughput transcriptomics also revealed that N limitations not only significantly altered the expression of the genes involved in the biosynthesis and endocytosis of nitrogenous macromolecules, but also led to the changes in the expression of genes involving photosynthesis, the tricarboxylic acid cycle and the pentose phosphate pathway (Fig. 2). Therefore, we assumed that the C/N balance is pivotal for maintaining the optimal growth of plants and enhancing the adaptability of plants to N limitations.

Previous studies have revealed that the *Arabidopsis* null mutant *atnla1* that fails to produce anthocyanins shows low-N-induced early senescence [3, 14, 34]. Under low N stresses, much lower survival rates combined with defects in anthocyanin accumulation are found in *A. thaliana* mutants (*myb75* and *dfr*) than in the wild type [35]. In this study, we also determined that numerous anthocyanin biosynthesis-related genes and *MYB* transcription factor genes were remarkably up-regulated under N depletion (Fig. 3). All of these findings indicated that the anthocyanin-dependent organic C metabolism may be crucial for the adaptability of plants to N limitations. Functioning as an E3 ubiquitin ligase, NLA1 degrades its target protein through the 26S proteome pathway [5]. Therefore, we assumed that the target protein that is degraded by NLA1 should be up-regulated in the *atnla1* null mutant, leading to the repression of anthocyanin biosynthesis. According to the criterion, we found that the expression of MYB2 (At2g47190), a transcriptional repressor of anthocyanin pigmentation [36], was increased by approximately 25-fold in *atnla1* [14], and it might be involved in the NLA1-mediated disruption of anthocyanin biosynthesis.

## Molecular characterization of the BnamiR827-*BnaNLA1*-*BnaNRT1.7* circuit

Ancient polyploidy events have been identified in the genomes of rapeseed progenitors, and duplicated regions of the *Arabidopsis* genome occur 10 to 14 times within the allotetraploid rapeseed genome (A_n_A_n_C_n_C_n_) [37]. The duplicated genes provide novel resources for the formation of new genes, which, in turn, contribute to gene loss, neo-functionalization and sub-functionalization [38]. Gene family members are both selected and preserved during the evolutionary process because they express variable levels of proteins in different spatiotemporal patterns [39].

In this study, we first conducted an integrated analysis of the BnamiR827-*BnaNLA1*-*BnaNRT1.7* regulatory circuit in the polyploidy crop species. Compared with that in the model *Arabidopsis* and rice, multi-copy homologs of both *NLAs* and *NRT1.7* were identified in the rapeseed genome (Additional file 1: Table S4, S5). For the BnaNLA proteins, the conserved motifs of SPX and RING were maintained (Additional file 1: Figure S3), and purifying selection occurrence of *BnaNLAs* (Additional file 1: Table S3) also highlighted their maintenance during the alloploidy process. However, significant divergence was observed in the function of *BnaNLAs* and *BnaNRT1.7 s* differing from that in the model plants.

In *Arabidopsis*, *NLA1* acting as an E3 ubiquitin ligase mediates the degradation of NRT1.7, and contributes to the efficient remobilisation of N from source to sink leaves; moreover, the expression of *AtNLA1* is not regulated by N supply at the transcriptional level [5]. However, in this study, both the qRT-PCR and RNA-seq results showed that *BnaNLA1s* were expressed dominantly in the roots rather than in the leaves (Fig. 5a, b); furthermore, the transcript levels of *BnaNLA1s* were repressed by N limitations (Fig. 5b). Based on these findings, we proposed that *NLA1s* might be mainly implicated in the root N remobilization in *B. napus*. Novel transcriptional mechanisms, regulated by the enriched Dof and WRKY transcription factors in the gene promoters (Fig. 5e), underlying *NLA1* regulation potentially existed. In addition, BnamiR827 was up-regulated by N limitations (Fig. 6d) and its target sites were observed in the *BnaNLA1* sequences (Fig. 6b, c), which was potentially involved in the post-transcriptional and translational repression of *BnaNLA1s*. AtNRT1.7 is expressed preferentially in the phloem of the leaf minor veins and mediates the remobilization of excess NO_3_^−^ from older leaves to younger ones [4]. Nonetheless, among the six *BnaNRT1.7* homologs, four were expressed dominantly in the shoots whereas the other two were expressed mainly in the roots (Fig. 7); they might be also involved in the root phloem N remobilization. Moreover, we also predicted several lysine amino acid residues as potential targets that were identified by the E3 ubiquitin ligase NLA1. Our findings suggested that, under N limitations, the involvement of BnamiR827-*BnaNLA1*-*BnaNRT1.7* regulatory circuit might be involved in leaf N remobilization as well as in the efficient re-translocation of root phloem N of rapeseed plants. Overall, during the allopolyploidy process, the BnamiR827-*BnaNLA1*-*BnaNRT1.7* not only maintained their intrinsic roles in NLA, but also developed a novel function in regulating efficient N metabolism.

### Proposed model of the molecular strategies involving N limitation adaptation in allotetraploid rapeseed

**Fig. 9:**
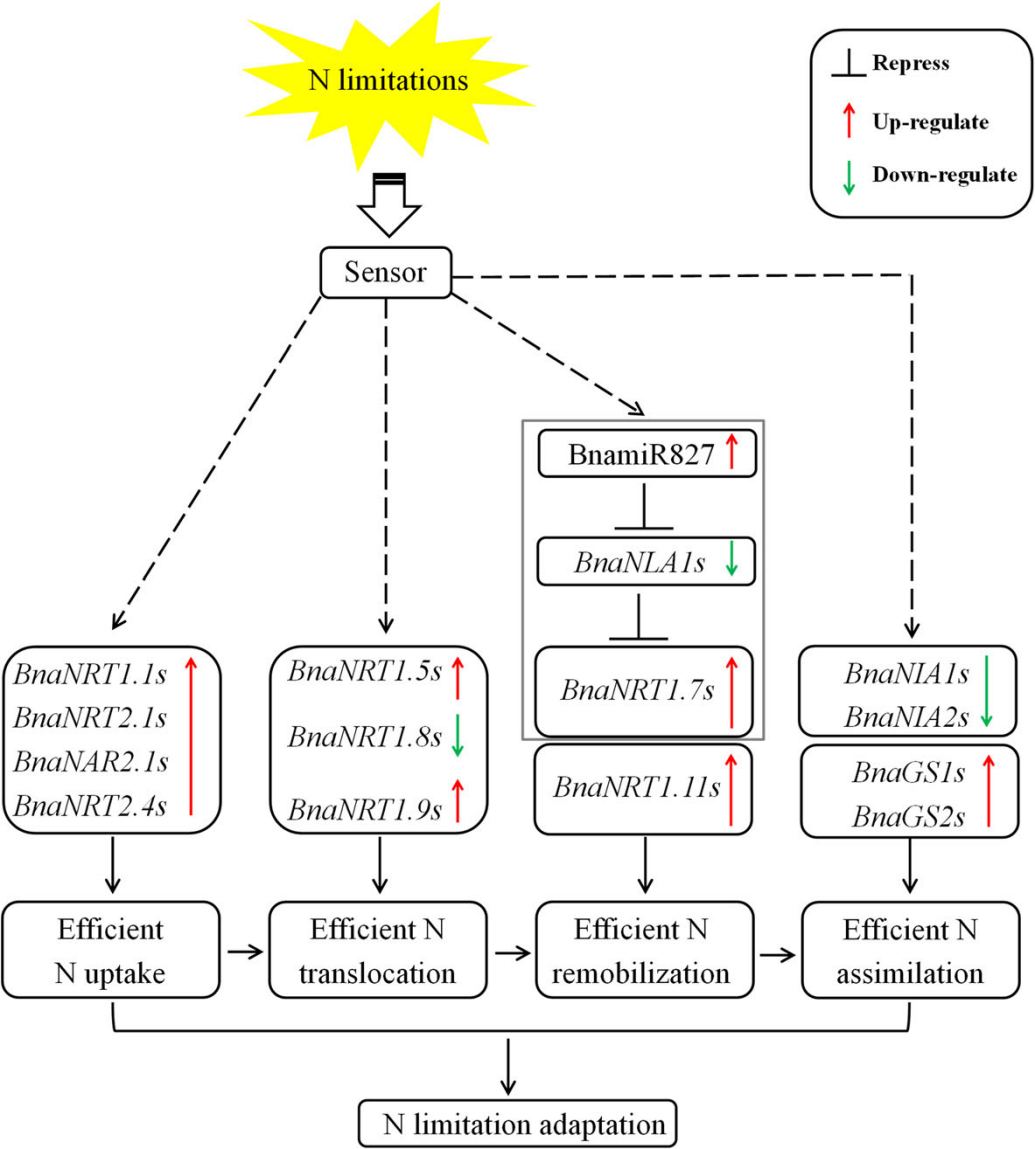
A proposed model for the adaptive strategies involving nitrogen (N) limitation adaptation in allotetraploid rapeseed. The dashed lines indicate potential or indirect regulation, and the red and green solid lines denote the up-regulation and down-regulation of gene expression induced by N limitations. The BnamiR827-*BnaNLA1*-*BnaNRT1.7* regulatory circuit is boxed by a rectangle

Under limited N stresses, plants usually develop a series of multifaceted adaptive responses, including physiologic, biochemical, transcriptional and proteomic alterations [40]. Based on the physiologic, genomic and transcriptional findings, we proposed a model to elucidate the molecular strategies that were used by rapeseed plants to enhance the NLA of plants (Fig. 9). Under N limitations, both the dual-affinity *BnaNRT1.1 s* and high-affinity *BnaNRT2.1 s* were up-regulated to increase root N uptake. Further, the increased N xylem loading co-regulated by *BnaNRT1.5 s* and *BnaNRT1.8 s* contributes to efficient N translocation to the shoots, fulfilling the N requirement for photosynthesis. In the shoots and roots, the induction of BnamiR827 repressed the expression of *BnaNLA1s*, and relieved the ubiquitin-mediated degradation of BnaNRT1.7 s, which is favorable for the efficient remobilization of N resources. Eventually, the enhanced activity of GS facilitated N assimilation to provide amino acids required for plant growth. Taken together, when under N deficiency stresses, the plants would develop a set of systematic responses involving efficient N uptake, translocation, remobilization and assimilation to enhance their adaptability to N limitations.

## Conclusions

In this study, we first made an integrated analysis of physiologic, genomic and transcriptional insights into the adaptive strategies of rapeseed plants to N limitations, and found numerous functional genes, in allotetraploid rapeseed, that diverged from those in the model Arabidopsis. The transcriptomics-assisted gene co-expression networks involving the genes that regulate N homeostasis provide central gene resources for the genetic improvement of crop NLA and NUE.

## Methods

### Quantification of chlorophyll, anthocyanin and N concentrations

The *B. napus* seedlings were hydroponically grown according to a randomized complete block design using the Hoagland solution, which was constantly aerated throughout the experiments and refreshed every 5 d [41]. The culture regimes of light and temperature were set as follows: 300–320 μmol m^− 2^ s^− 1^; 24 °C daytime/22 °C night; 16 h photoperiod).

For the NO_3_^−^-depletion treatments, the rapeseed seedlings of the cultivar “Xiang-you 15” (“XY15”) that were hydroponically grown under high NO_3_^−^ (9.0 mM) for 10 d were then transferred to low NO_3_^−^ (0.30 mM). At 0 h, 3 h and 72 h, the shoots and roots of the “XY15” seedlings were individually sampled. The SPAD values of older leaves were measured using a SPAD-502 Chlorophyll Meter (Konica Minolta, Tokyo, Japan). The anthocyanin concentration in the leaves of rapeseed seedlings was assayed according to the method described by Mancinelli et al [42] N metabolism in plants is tightly linked to the activity of several key enzymes, such as nitrate reductase (NR, EC 1.7.99.4) and glutamine synthetase (GS, EC 6.3.1.2) [43]. For NR activity determination, the fresh roots and leaves were ground to fine powder (~ 100 mg), and then were extracted and determined spectrophotometrically as described by Ehlting et al. [44]. The glutamine synthetase activity was assayed with the method reported by Wang et al. [45]. The NO_3_^−^ concentrations in the roots and leaves of rapeseed plants were determined spectrophotometrically at 410 nm according to Patterson et al [46] Total N concentrations of rapeseed were assayed with the method reported by Wang et al [47] In this study, NUE = total biomass/total N accumulation according to Li et al. [48].

To identify natural variations in the adaptabilities of rapeseed genotypes to N limitation, we subjected a panel that comprises 102 accessions to hydroponic culture. The rapeseed plants that were grown under sufficient (9.0 mM) NO_3_^−^ for 10 d were then transferred to limited N (0.3 mM NO_3_^−^) supply for 5 d, which was used for the assessment of low-N tolerance based on the SPAD values.

In this study, all the seeds of rapeseed plants were obtained from the research group led by Prof. Chun-yun Guan (Hunan Agricultural University, Hunan Province, China).

### Transcriptional characterization of rapeseed responses to N limitations

Regarding the NO_3_^−^-depletion treatments, the seedlings of the rapeseed cultivar, “XY15”, that were hydroponically grown under high NO_3_^−^ (9.0 mM) for 10 d were then transferred to low NO_3_^−^ (0.30 mM). After exposure to low NO_3_^−^ for 0 h, 3 h and 72 h, the shoots and roots of the “XY15” seedlings were individually sampled, and a total of 18 tissue samples were collected for mRNA sequencing (RNA-seq). Regarding the NO_3_^−^ resupply treatments, the “XY15” seedlings that were hydroponically grown under high NO_3_^−^ (9.0 mM) for 9 d were transferred to NO_3_^−^-free solution for 3 d. The seedlings were sampled after supplied with 9.0 mM NO_3_^−^ for 6 h, and 12 samples were collected for RNA-seq analysis.

The leaves and roots of rapeseed seedlings above-mentioned were individually harvested, and three independent biological replicates for each tissue. Total RNA, which was isolated using the pre-chilled RNAiso plus (Takara Bio Inc., Kusatsu, Shiga, Japan), were subjected to the assessment of RNA integrity number (RIN). A total of 30 RNA samples (~ 2.0 μg) with the RIN values > 8.0 were used to construct strand-specific cDNA libraries, which were used for the high-throughput transcriptomic sequencing on a lane of an Illumina Hiseq 4000 platform (read length = 150 bp, paired end). The gene expression were normalized using the Fragments Per Kilobase of exon model per Million mapped reads (FPKM) values, and the criteria for false discovery rate ≤ 0.05 and absolute values of log_2_(fold-change) ≥1 were used to characterize gene differential expression [49]. Analyses of gene ontology (GO) and metabolic route enrichment for the differentially expressed genes (DEGs) were performed using PANTHER (http://www.pantherdb.org/) [50] and Kyoto Encyclopedia of Genes and Genomes (KEGG) (http://www.kegg.jp/) [51], respectively. Heat maps that show gene expression profiling were delineated by Multiexperiment Viewer (Mev, http://www.mybiosoftware.com/mev-4-6-2-multiple-experiment-viewer.html) [52]. We established gene co-expression networks using CYTOSCAPE v. 3.2.1 (http://www.cytoscape.org/) [53], which were used to characterize the core genes involving the response of oilseed rape to N limitation. For each gene pair, the *Pearson* coefficient threshold was set based on the defaults (http://plantgrn.noble.org/DeGNServer/Analysis.jsp).

### Retrieval of genomic, coding and amino acid sequences of target genes

The Ath-MIR827 (At3g59884), *AtNLA1* (At1g02860), *AtNLA2* (At2g38920) and *AtNRT1.7/AtNPF2.13* (At1g69870) gene sequences were used as the seed sequences, and BLASTn and BLASTp analyses were conducted to search the homolog sequences in *B. rapa*, *B. oleracea*, *B. napus* and other plant species. The databases used in this study included TAIR (https://www.arabidopsis.org/) for *A. thaliana*, the *Brassica* Database v. 1.1 (http://brassicadb.org/brad/) [9, 54], *EnsemblPlants* (http://plants.ensembl.org/Brassica_oleracea/Info/Index), NCBI (www.ncbi.nlm.nih.gov) and Phytozome v. 10 (http://phytozome.jgi.doe.gov/pz/portal.html) [55]. InterProScan5 (http://www.ebi.ac.uk/interpro/search/sequence-search) [56] and the conserved domain database (http://www.ncbi.nlm.nih.gov/Structure/bwrpsb/bwrpsb.cgi) [57] were used to determine the absence/presence of the SPX (Pfam PF03105) and RING motifs (PLN00028).

### Multiple sequence alignment and phylogeny analysis

Full-length sequences of proteins were aligned using Clustal W within Molecular Evolutionary Genetics Analysis (MEGA) v. 7.0.26 (http://www.megasoftware.net/) [58]. After these alignments, the phylogenetic trees were constructed with the neighbor-joining method [59]. Complete deletion was used for the analysis of sequence gaps and missing data, and the *Poisson* correction model was used to compute the phylogeny distance. We conducted a bootstrap analysis with 1,000 replicates to examine the statistical reliability of the phylogeny relationships and nodes with a bootstrap threshold value of 50%. The structural divergence among the proteins in *A. thaliana* and *Brassica* crops was determined by subjecting the full-length sequences of amino acids to the Multiple Em Motif Elicitation (MEME) online program v. 4.12.0 (http://meme-suite.org/tools/meme) [60] to characterize the conserved motifs/domains with the default parameters.

### Physio-chemical characterization of the NLA and NRT1.7 proteins

ExPASy ProtoParam (http://www.expasy.org/tools/protparam.html) was used to identify the amino acid number and composition, molecular weight (MW, KD), theoretical isoelectric point (pI), grand average of hydropathy (GRAVY), and instability index of the NLA proteins. An instability index > 40 indicates that the protein is unstable. WoLF PSORT (http://www.genscript.com/wolf-psort.html) [61] was used to predict the subcellular localisation of the NLA and NRT1.7 proteins. We subjected the amino acid sequences to TMpred (https://embnet.vital-it.ch/software/TMPRED_form.html) [62] for the characterization of membrane-spanning regions and orientations.

### Elucidation of protein ubiquitin sites and microRNA target sites

The target sites of the *NLA* family genes recognised by microRNAs were predicted using psRNATarget v. 2017 (http://plantgrn.noble.org/psRNATarget/analysis?function=2) [31]. The mature sequence of BnamiR827 and its 200-bp flanking genomic sequences extending from each side were folded by using RNAFOLD v.2.2.9 [63]. The ubiquitin sites of the BnaNRT1.7 proteins were predicted by UbPred: predictor of protein ubiquitination sites (http://www.ubpred.org/) [64].

### Analysis of evolutionary selection pressure and functional divergence

The rates of synonymous (Ks) and non-synonymous (Ka) nucleotide substitution, and Ka/Ks were calculated to identify positive or negative (purifying) selection during the gene evolution process. Pairwise alignment of the gene coding sequences was performed using Clustal W (http://www.clustal.org/clustal2/) [65], and then the readout was subjected to KaKs_Calculator (https://sourceforge.net/projects/kakscalculator2/) [66] to calculate the Ka, Ks, and Ka/Ks with the yn00 method [67]. Generally, Ka/Ks > 1.0 denotes the occurrence of positive selection, while Ka/Ks < 1.0 indicates purifying selection, and Ka/Ks = 1 indicates neutral selection [68]. The formula T = Ks/2λ (λ = 1.5 × 10^− 8^ for Brassicaceae) [69] was used to judge the time of gene divergence.

Gene functional divergence between the *NLA1* and *NLA2* clusters was estimated using DIVERGE v. 3.0 (http://xungulab.com/software/diverge3/diverge3.html) [70]. The level of type-II functional divergence is referred to as the θ_II_ coefficient. If θ_II_ = 0, it indicates no type-II functional divergence; however, θ_II_ = 1 shows a very significant divergence.

### Identification of putative *cis*-acting regulatory elements (CREs) in the gene promoters

For each gene, a 2.0-kb genomic sequence upstream from the start codon (ATG) was downloaded from the *B. napus* Genome Browser (http://www.genoscope.cns.fr/brassicanapus/) [9]. These sequences were subjected to plantCARE (http://bioinformatics.psb.ugent.be/webtools/plantcare/html/) [71] to identify putative CREs, which were illustrated using the word cloud generator WordArt (https://wordart.com/).

### Quantitative reverse-transcription PCR (qRT-PCR) assays

After treatment of RNA samples with RNase-free DNase I, the total RNA was used as the templates for cDNA synthesis with the PrimeScript™ RT reagent Kit with gDNA Eraser (Perfect Real Time) (TaKaRa, Shiga, Japan). The stem-loop reverse transcription PCR was used in the qRT-PCR experiments for miR827 according to Chen et al. [72], and the BnaU6 small nuclear RNA (snRNA) was used as an internal control for each reaction [73]. The following primers were used for reverse transcription of BnamiR827: GTCGTATCCAGTGCAGGGTCCGAGGTATTCGCACTGGATACGACTATTTG, and the *BnaU6* snRNA was reverse transcribed using random primers.

The qRT-PCR assays for the detection of relative gene expression were performed using SYBR® *Premix Ex Taq*™ II (Tli RNaseH Plus) (TaKaRa, Shiga, Japan) under an Applied Biosystems StepOne™ Plus Real-time PCR System (Thermo Fisher Scientific, Waltham, MA, USA). The thermal cycles were as follows: 95 °C for 3 min, followed by 40 cycles of 95 °C for 10 s, and 60 °C for 30 s. The melt curve analysis was conducted as follows to ensure the primer gene-specificity: 95 °C for 15 s, 60 °C for 1 min, and 60–95 °C for 15 s (+ 0.3 °C per cycle). Expression data of the *BnaNLA* and *BnaNRT1.7* family genes were normalized using the public reference genes *BnaEF1-α* [74] and *BnaGDI1* [75], and the relative gene expression was calculated with the 2^-ΔΔC^_*T*_ method [76]. The gene-specific primers of BnamiR827, *BnaNLAs* and *BnaNRT1.7 s* for qRT-PCR assays are listed in Additional file 1: Table S1.

### Statistical analysis

For statistical tests, the significant difference was determined by one-way analysis of variance (ANOVA), which was followed by Tukey’s honestly significant difference multiple comparison tests using the Statistical Productions and Service Solutions 17.0 (SPSS, Chicago, IL, USA).

## Additional file

Additional file 1: **Table S1.** Gene-specific Primers used for qRT-PCR assays in this study. **Table S2.** Overview of the high-throughput RNA-seq data of the short-term and long-term nitrogen limitation experiments. **Table S3.** Evolutionary selection pressure analysis of the *BnaNLA* family genes. **Table S4.** Molecular characterization of the BnaNLA family proteins in *Brassica napus*. **Table S5.** Molecular characterization of the BnaNRT1.7/BnaNPF2.13 family proteins in *Brassica napus*. **Figure S1.** Copy number of the *NLA1* and *NLA2* family genes in diverse plants species. **Figure S2.** Hypothetical evolutionary processes and expansion events of the *NLA* family genes in *A. thaliana* and *Brassica* crops. **Figure S3.** Analysis of phylogenetic relationships and functional divergence of the NLA proteins. **Figure S4.** Molecular characterization of the expression pattern of *BnaNLA2s* under different N supply. **Figure S5.** Hypothetical evolutionary processes and expansion events of the *NRT1.7* family genes in *A. thaliana* and *Brassica* crops. **Figure S6.** Analysis of phylogenetic relationships and conserved motifs of the NRT1.7 proteins. (DOCX 855 kb)

## Abbreviations

BLAST: Basic local alignment search tool
CNV: Copy number variation
CRE: *cis*-acting regulatory elements
DEG: Differentially expressed gene
GS: Glutamine synthetase
miRNA: microRNA
MYA: Million years ago
N: Nitrogen
NLA: Nitrogen limitation adaptation
NO_3_^−^: Nitrate
NPF: Nitrate/peptide family
NR: Nitrate reductase
NRT: Nitrate transporter
NUE: Nitrogen use efficiency
PAV: Presence/absence variation
qRT-PCR: quantitative reverse-transcription polymerase chain reaction
