# Supplementary material for: Integrated physiologic, genomic and transcriptomic strategies involving the adaptation of allotetraploid rapeseed to nitrogen limitation
Source: BMC Plant Biol. 2018 Dec 4;18:322. doi: 10.1186/s12870-018-1507-y (PMC6278020; doi:10.1186/s12870-018-1507-y)
Supplement: Supplementary file 1 — Table S1. Gene-specific Primers used for qRT-PCR assays in this study. Table S2. Overview of the high-throughput RNA-seq data of the short-term and long-term nitrogen limitation experiments. Table S3. Evolutionary selection pressure analysis of the BnaNLA family genes. Table S4. Molecular characterization of the BnaNLA family proteins in Brassica napus. Table S5. Molecular characterization of the BnaNRT1.7/BnaNPF2.13 family proteins in Brassica napus. Figure S1. Copy number of the NLA1 and NLA2 family genes in diverse plants species. Figure S2. Hypothetical evolutionary processes and expansion events of the NLA family genes in A. thaliana and Brassica crops. Figure S3. Analysis of phylogenetic relationships and functional divergence of the NLA proteins. Figure S4. Molecular characterization of the expression pattern of BnaNLA2s under different N supply. Figure S5. Hypothetical evolutionary processes and expansion events of the NRT1.7 family genes in A. thaliana and Brassica crops. Figure S6. Analysis of phylogenetic relationships and conserved motifs of the NRT1.7 proteins. (DOCX 855 kb) [file 12870_2018_1507_MOESM1_ESM.docx]

**Additional files**

**Additional file 1**

**Figure S1** Copy number of the *NLA1* and *NLA2* family genes in diverse plants species. The dicot species include *Amborella trichopoda* (genome size: 748 Mb), *Arabidopsis lyrata* (207 Mb), *Arabidopsis thaliana* (125 Mb), *Brassica napus* (1,345 Mb), *Brassica oleracea* (630 Mb), *Brassica rapa* (485 Mb), *Carica papaya* (370 Mb), *Citrullus lanatus* (425 Mb), *Citrus sinensis* (367 Mb), *Cucumis sativus* (350 Mb), *Glycine max* (1,100Mb), *Medicago truncatula* (500 Mb), *Musa acuminate* (523 Mb), *Populus trichocarpa* (480 Mb), *Prunus persica* (265 Mb), *Solanum lycopersicum* (900 Mb), *Solanum tuberosum* (844 Mb), *Theobroma cacao* (430 Mb) and *Vitis vinifera* (490 Mb). The monocot species include *Oryza sativa* (466 Mb), *Sorghum bicolor* (730 Mb) and *Zea mays* (2,300 Mb).

**Figure S2** Hypothetical evolutionary processes and expansion events of the *NLA* family genes in *A. thaliana* and *Brassica* crops. Evolutionary processes and expansion events of the *NLA1* (A) and *NLA2* (B) genes of *A. thaliana*, *B. rapa*, *B. oleracea* and *B. napus*. The homologues between different *Brassica* species are connected by lines.

**Figure S3** Analysis of phylogenetic relationships and functional divergence of the NLA proteins. (A) Phylogeny analysis of the NLA proteins in Brassica species, including *Arabidopsis thaliana*, *Brassica rapa*, *Brassica oleraceae* and *Brassica napus*. (B) Functional divergence analysis of the NLA1 and NLA2 proteins in *Brassica napus*. The column indicates the coefficient (θ_II_) of type II functional divergence, and the error bar denotes the standard error. (C) Phylogenetic relationships of the NLA1 proteins in dicot and monocot species. AtNLA1 and BnaNLA1s are indicated by solid red circles. Poisson correction, pairwise deletion, and bootstrapping (1,000 replicates; random seeds) were set as the required parameters. (D) Identification and characterization of the ten most conserved motifs (1-10) of the BnaNLA proteins in *Arabidopsis* and *Brassica napus*. The SYG1-PHO81-XPR1 (SPX) and Really Interesting New Gene (RING) motifs are boxed by dashed rectangles. (E) Sequence characterization of the conserved motifs (M1-M10) in the BnaNLA proteins, which are presented by Weblogo (http://weblogo.berkeley.edu/logo.cgi). The larger the fonts, the more conserved the motifs.

**Figure S4** Molecular characterization of the expression pattern of *BnaNLA2s* under different N supply. (A) The qRT-PCR assay results showing the expression pattern of *BnaNLA2s*. (B-C) Transcriptional profiling of *BnaNLA2s* to N limitations (B) and N resupply (C). Regarding the NO_3_^-^-depletion treatments, the rapeseed seedlings that were cultivated under high NO_3_^-^ (9.0 mM) for 10 d were then transferred to low NO_3_^-^ (0.30 mM). At 0 h, 3 h and 72 h, the shoots and roots of the seedlings were individually sampled. Regarding the NO_3_^-^ resupply treatments, the *B. napus* seedlings that were hydroponically cultivated under high NO_3_^-^ (9.0 mM) for 9 d were then transferred to NO_3_^-^-free solution for 3 d. The seedlings were sampled after being treated with 9.0 mM NO_3_^-^ for 6 h, respectively.

**Figure S5** Hypothetical evolutionary processes and expansion events of the *NRT1.7* family genes in *A. thaliana* and *Brassica* crops. Evolutionary processes and expansion events of the *NRT1.7* genes of *A. thaliana*, *B. rapa*, *B. oleracea* and *B. napus*. The homologues between different *Brassica* species are connected by lines.

**Figure S6** Analysis of phylogenetic relationships and conserved motifs of the NRT1.7 proteins. (A) Phylogeny analysis of the NRT1.7 proteins in Brassica species, including *Arabidopsis thaliana*, *Brassica rapa*, *Brassica oleraceae* and *Brassica napus*. Poisson correction, pairwise deletion, and bootstrapping (1,000 replicates; random seeds) were set as the required parameters. (B) Evolutionary selection pressure and divergence time of *BnaNRT1.7s*. (C) Identification and characterization of the ten most conserved motifs (1-10) of the BnaNRT1.7 proteins in *Arabidopsis* and *Brassica napus*. (D) Sequence characterization of the conserved motifs (M1-M10) in the BnaNRT1.7 proteins, which are presented by Weblogo (http://weblogo.berkeley.edu/logo.cgi). The larger the fonts, the more conserved the MFS/NNP motifs.

**Table S1** Gene-specific Primers used for qRT-PCR assays in this study

| Gene name | Forward (5’-3’) | Reverse (5’-3’) | Amplification efficiency |
| --- | --- | --- | --- |
| *BnaC5.NLA1* | TGAAAACTGCAGACCCGTCC | TCTTCCCAGTACTCTCGGCG | 93.6% |
| *BnaA9.NLA1* | CAACTGTCCTCGTGAATGCG | GATCAAACCAAGATGATGGCTC | 98.1% |
| *BnaA10.NLA1* | CATTTAGCTTCTCGTTTCAAAAGG | CGAGTTGATGAGGGCGTAGC | 96.5% |
| *BnaC8.NLA1* | TGCAGAAGAAGAGATCATCTTCCTC | CTCGTTTTTGAATGATTGCCTC | 94.9% |
| *BnaA3.NLA2* | CATGTAACACCACAAGATCTGATGG | GAAACTTCCTTCATCAGCTCCG | 95.7% |
| *BnaC3.NLA2* | CCACAATGTTCCAAATGTCCC | ATCTCAGACCGTTCCCCCAC | 101.1% |
| *BnaA4.NLA2* | CAATCCCTATGCTTTGAAGTGC | GGACATTTGGAACATTGTGGG | 97.6% |
| *BnaC4.NLA2* | CAAGACATGTAACTCCAAAAGATCG | CTTGAAGCATCCTGCTATGTCG | 91.8% |
| *BnaA7.NRT1.7a* | AACGAACCGAGGAAGGGATC | CTGACTTGGTCTTGGATATACACG | 97.8% |
| *BnaC6.NRT1.7a* | AGGAAGGGATTAAAGGAGTAGCC | CTGACTTGGTCTTGGATATACAC | 99.4% |
| *BnaC6.NRT1.7b* | CAGCAACATGCATTAGCAAAGAC | CAAAAGGGATGCTACATGGC | 92.9% |
| *BnaA7.NRT1.7b* | GTTATTTTTTTCGCTGGAATGAG | AACTTGTGTAAAACGCTATCTTTAACG | 97.4% |
| *BnaA2.NRT1.7* | CGGGATGACTCCCATGTCG | GATGCTTCTCATGTGTTCAGGG | 96.7% |
| *BnaCn.NRT1.7* | GCTGGATCATCGGCTTTAGC | GAGCTTCATCTTTCGCTTCTTG | 99.7% |
| *BnaEF1-α* | GCCTGGTATGGTTGTGACCT | GAAGTTAGCAGCACCCTTGG | 100.0% |
| *BnaGDI* | GAGTCCCTTGCTCGTTTCC | TGGCAGTCTCTCCCTCAGAT | 93.1% |
|  |  |  |  |

**Table S2** Overview of the high-throughput RNA-seq data of the nitrogen limitation experiments

| Sample name | | Raw reads | Clean reads | Clean bases (G) | Error rate (%) | Q20  (%) | Q30  (%) | GC content  (%) | Mapped reads  (%) |
| --- | --- | --- | --- | --- | --- | --- | --- | --- | --- |
| Shoot | 0 h | 59,635,738 | 57,043,591 | 8.55 | 0.01 | 97.1 | 92.6 | 47.5 | 89.6 |
|  | 3 h | 58,163,929 | 55,612,390 | 8.34 | 0.02 | 97.2 | 92.8 | 47.6 | 89.8 |
|  | 72 h | 55,942,618 | 53,982,678 | 8.10 | 0.02 | 96.7 | 91.6 | 47.1 | 89.9 |
| Root | 0 h | 49,108,675 | 47,000,152 | 7.05 | 0.02 | 97.1 | 92.8 | 46.1 | 86.1 |
|  | 3 h | 44,434,570 | 42,606,356 | 6.39 | 0.02 | 96.9 | 92.2 | 45.6 | 87.3 |
|  | 72 h | 50,080,180 | 48,523,315 | 7.28 | 0.02 | 97.0 | 92.5 | 46.3 | 87.5 |

**Table S3** Evolutionary selection pressure analysis of the *BnaNLA* family genes

| Gene name | | Ka | Ks | Ka/Ks | Date (Mya)  T= Ks/2λ | Selective  pressure | Duplicated  type |
| --- | --- | --- | --- | --- | --- | --- | --- |
| *NLA1* | *BnaA9.NLA1* | 0.75 | 1.77 | 0.43 | 59.02 | Negative | Segmental |
|  | *BnaA10.NLA1* | 0.08 | 0.48 | 0.16 | 16.14 | Negative | Segmental |
|  | *BnaC5.NLA1* | 0.07 | 0.48 | 0.15 | 16.06 | Negative | Segmental |
|  | *BnaC8.NLA1* | 0.08 | 0.42 | 0.18 | 14.15 | Negative | Segmental |
| *NLA2* | *BnaA3.NLA2* | 0.07 | 0.39 | 0.17 | 12.83 | Negative | Segmental |
|  | *BnaA4.NLA2* | 0.06 | 0.41 | 0.14 | 13.77 | Negative | Segmental |
|  | *BnaC3.NLA2* | 0.06 | 0.44 | 0.15 | 14.57 | Negative | Segmental |
|  | *BnaC4.NLA2* | 0.05 | 0.41 | 0.13 | 13.75 | Negative | Segmental |

Note: λ=1.5*10^-8^

**Table S4** Molecular characterization of the BnaNLA family proteins in *Brassica napus*

|  | Gene name | Gene ID | Block | Subgenome | CDS (bp) | Amino acids | MW (kD) | pI | Instablility index | GRAVY |
| --- | --- | --- | --- | --- | --- | --- | --- | --- | --- | --- |
| *NLA1* | *BnaA9.NLA1* | BnaA09g51130D | A | LF | 996 | 331 | 37.9 | 8.49 | 54.2 | -0.386 |
|  | *BnaA10.NLA1* | BnaA10g01450D | A | LF | 1002 | 333 | 38.2 | 8.48 | 38.4 | -0.421 |
|  | *BnaC5.NLA1* | BnaC05g01480D | A | LF | 999 | 332 | 38.1 | 8.83 | 50.4 | -0.413 |
|  | *BnaC8.NLA1* | BnaC08g45940D | A | LF | 993 | 330 | 37.9 | 8.65 | 50.1 | -0.395 |
| *NLA2* | *BnaA3.NLA2* | BnaA03g18240D | J | MF2 | 1002 | 333 | 38.6 | 8.61 | 42.6 | -0.253 |
|  | *BnaA4.NLA2* | BnaA04g22260D | J | MF1 | 1324 | 661 | 75.6 | 6.17 | 47.7 | -0.233 |
|  | *BnaC3.NLA2* | BnaC03g72250D | J | MF2 | 1002 | 333 | 38.8 | 8.52 | 41.5 | -0.274 |
|  | *BnaC4.NLA2* | BnaC04g46040D | J | MF1 | 1509 | 333 | 38.8 | 8.62 | 45.7 | -0.266 |

Note: CDS, coding sequence; MW, molecular weight; pI, isoelectric point; GRAVY, grand average of hydropathicity; TM, transmembrane.

**Table S5** Molecular characterization of the BnaNRT1.7/BnaNPF2.13 family proteins in *Brassica napus*

| Gene name | Gene ID | Block | Subgenome | CDS (bp) | Amino acids | MW (kD) | pI | Instablility index | GRAVY | TM |
| --- | --- | --- | --- | --- | --- | --- | --- | --- | --- | --- |
| *BnaA2.NRT1.7* | BnaA02g35730D | E | MF1 | 1776 | 591 | 65.3 | 8.45 | 38.2 | 0.271 | 12 |
| *BnaA7.NRT1.7a* | BnaA07g28390D | E | LF | 1869 | 622 | 68.7 | 9.09 | 39.6 | 0.190 | 12 |
| *BnaA7.NRT1.7b* | BnaA07g24080D | E | LF | 1824 | 607 | 67.4 | 8.95 | 39.4 | 0.237 | 12 |
| *BnaC6.NRT1.7a* | BnaC06g30920D | E | LF | 1860 | 619 | 68.5 | 9.10 | 39.4 | 0.190 | 12 |
| *BnaC6.NRT1.7b* | BnaC06g25140D | E | LF | 1824 | 607 | 67.3 | 9.02 | 37.0 | 0.224 | 13 |
| *BnaCn.NRT1.7* | BnaCnng24110D | E | MF1 | 1785 | 594 | 65.7 | 8.81 | 37.0 | 0.268 | 12 |

Note: CDS, coding sequence; MW, molecular weight; pI, isoelectric point; GRAVY, grand average of hydropathicity; TM, transmembrane.
